# Zn(II) to Ag(I) Swap in Rad50 Zinc Hook Domain Leads to Interprotein Complex Disruption through the Formation of Highly Stable Ag*_x_*(Cys)*_y_* Cores

**DOI:** 10.1021/acs.inorgchem.2c03767

**Published:** 2023-03-02

**Authors:** Olga Kerber, Józef Tran, Alicja Misiaszek, Aleksandra Chorążewska, Wojciech Bal, Artur Krężel

**Affiliations:** † Department of Chemical Biology, Faculty of Biotechnology, University of Wrocław, Joliot-Curie 14a, 50-383 Wrocław, Poland; ‡ Institute of Biochemistry and Biophysics, Polish Academy of Sciences, Pawińskiego 5a, 02-106 Warsaw, Poland

## Abstract

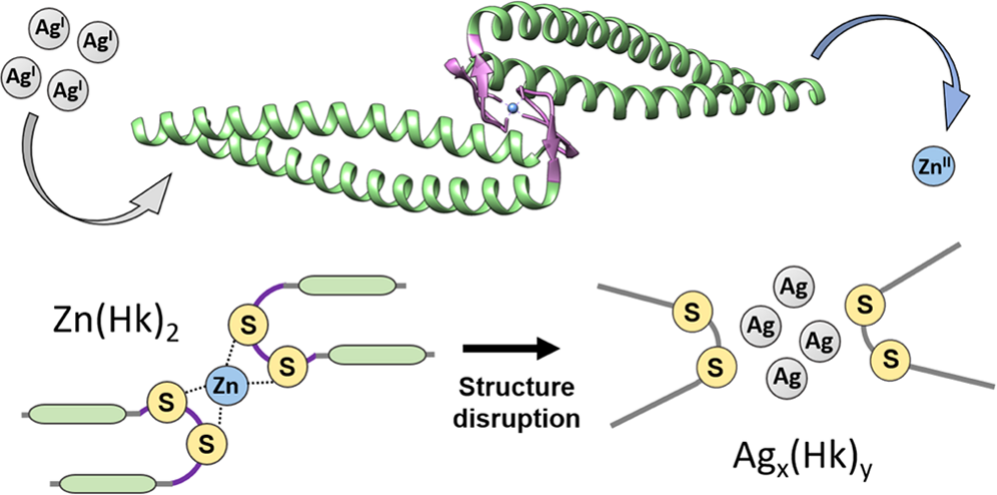

The widespread application of silver nanoparticles in medicinal and daily life products increases the exposure to Ag(I) of thiol-rich biological environments, which help control the cellular metallome. A displacement of native metal cofactors from their cognate protein sites is a known phenomenon for carcinogenic and otherwise toxic metal ions. Here, we examined the interaction of Ag(I) with the peptide model of the interprotein zinc hook (Hk) domain of Rad50 protein from *Pyrococcus furiosus*, a key player in DNA double-strand break (DSB) repair. The binding of Ag(I) to 14 and 45 amino acid long peptide models of apo- and Zn(Hk)_2_ was experimentally investigated by UV–vis spectroscopy, circular dichroism, isothermal titration calorimetry, and mass spectrometry. The Ag(I) binding to the Hk domain was found to disrupt its structure via the replacement of the structural Zn(II) ion by multinuclear Ag*_x_*(Cys)*_y_* complexes. The ITC analysis indicated that the formed Ag(I)–Hk species are at least 5 orders of magnitude stronger than the otherwise extremely stable native Zn(Hk)_2_ domain. These results show that Ag(I) ions may easily disrupt the interprotein zinc binding sites as an element of silver toxicity at the cellular level.

## Abstract 2

We examined the interaction of Ag(I) with the peptide model of the interprotein zinc hook (Hk) domain of Rad50 protein from *Pyrococcus furiosus*, a key player in DNA double-strand break (DSB) repair.

## Introduction

Metallic silver Ag(0) and Ag(I) compounds have a long history of use as antimicrobial and antifungal agents owing to their unique biocidal properties.^1^ In recent years, metallic silver-based biocides gained immense popularity in topical applications due to widespread antibiotic resistance.^2,3^ Since these biocides are not regulated, they have also become extensively used beyond medicine, including food preservation and a vast range of consumer goods, such as cosmetics, clothes, and more.^4^ Silver metal nanoparticles (AgNPs) are most common in these applications due to their favorable activity stemming from a high surface-to-volume ratio. The biocidal activity of AgNPs arises mainly from their oxidative dissolution in biological environments and subsequent release of the actual bioactive Ag(I) species.^5,6^ Such species are then able to induce ROS production and form dysfunctional complexes with peptides, proteins, and DNA.^7−11^ The expanding use of AgNPs raised concern about the increased bioavailability of silver, as the high mobility of NPs inside the body may lead to silver accumulation and toxicity in organs distant from the site of contact, including the liver.^12−17^ Detailed studies of Ag(I) speciation and its interaction with biomolecules, such as binding to different protein motifs, are therefore critically important to assess and minimize the potential hazards of AgNP use.

In biological systems, cysteine residues and low-molecular-weight (LMW) thiols, such as glutathione, are found to be common targets of Ag(I).^6,8,17,18^ This is not surprising. Due to a strong preference toward soft bases, Ag(I) ions display a very high affinity toward thiol ligands. For example, *K*_1_ for the Ag(I) complex of cysteine is more than 8 orders of magnitude more stable than that of methionine and nearly 10 orders stronger than that of histidine (log *K*_1_ of 11.9 vs 3.29 vs 2.5).^19−21^ Solid-state and solution studies of LMW Ag(I)–thiolate structures revealed a strong tendency of thiolate sulfur to bridge Ag(I) ions in a two- and three-coordinate manner, demonstrating either linear AgS_2_ or trigonal planar AgS_3_ geometry, respectively.^22,23^ These studies also evidenced that the architecture of Ag(I)–thiolate complexes is determined by the steric hindrances between the ligands.^23^ Accordingly, the steric effects are likely the important factor driving the organization of Ag(I)–thiolate sites inside the crowded protein scaffolds. In fact, Ag(I) is known for its relatively flexible coordination sphere, not constrained by d-orbital directionality, due to the spherical symmetry of the filled-shell d^10^ configuration.^24^ Such flexibility allows the ion to be coordinated by a variety of ligands and to adopt various geometries. The coordination sphere of Ag(I) in proteins is, therefore, often completed by noncysteine ligands, such as imidazole nitrogen or methionine sulfur, leading to the adoption of various coordination numbers (generally 2–4) and geometries by the Ag(I) ion.^25^ Nevertheless, in structures where the Ag(I)–thiolate bonding dominates, Ag(Cys)_2_ and Ag(Cys)_3_ coordination spheres are mostly formed, with the characteristic bond lengths of 2.40 and 2.49 Å, respectively.^26^ Structural examples of Ag(I)–thiolate sites demonstrate the formation of mono- (PDB ID: 1Q06,^27^5NXL,^28^6XTL,^29^2MZC^30^) and multinuclear (PDB ID: 1AOO,^31^5F0W^32^) centers, localized either within a single polypeptide chain^27,28,31^ or at protein interfaces.^30,32^ Such diversity of binding architectures reflects the mentioned plasticity of Ag(I)–thiolate sites, which enables them to accommodate different protein conformations and may also relate to the Ag(I) binding strength.

**Figure 1:**
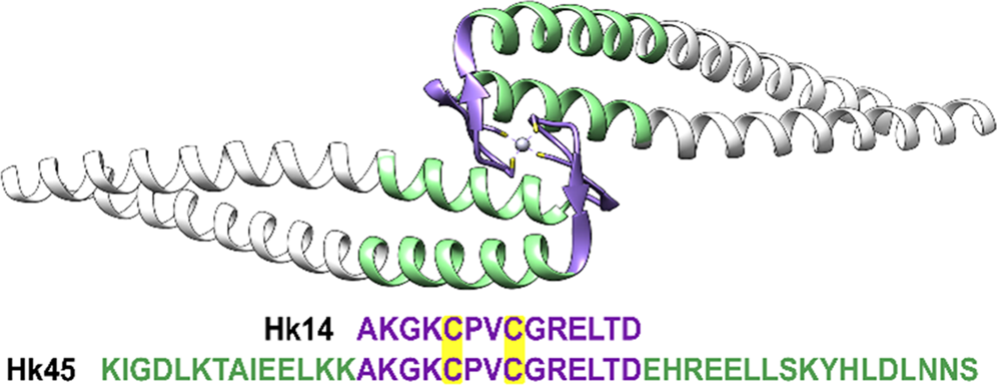
Crystal structure representation of the *P. furiosus* Rad50 coiled-coil zinc hook dimer (396–498) (PDB: 1L8D)^43^ with marked hook domain model fragments^45−48^ used in this study: Hk14 (440–453, violet) and Hk45 (426–470, green + violet).

The binding of Ag(I) may abrogate the protein structure dynamics,^33^ prevent the formation of the enzyme–substrate complex through the catalytic site blockage,^25^ or compete for the physiological metal ions’ cognate sites.^11,26,34−37^ This leads to protein malfunction and metal dyshomeostasis. Structural and catalytic effects are interwoven in cases when the native metal ion modulates protein structure stability and dynamics.^36,37^ Recently, we demonstrated a spontaneous Zn(II) to Ag(I) swap in the main variants (CCHH, CCCH/CCHC, and CCCC) of the consensus peptide-1 zinc finger (ZF).^36^ Substituting for Zn(II) ions, Ag(I) ions destroyed the native ZF fold in the course of cooperative formation of Ag*_n_*(Cys)*_n_* clusters that were saturated at 1:1 Ag(I)/Cys stoichiometry. In the following *in vitro* study, we also showed that the Zn(II)-to-Ag(I) exchange in a ZF protein derived from the Sp1 transcription factor (PDB ID: 1MEY) resulted in a loss of structure and dissociation of the protein from its cognate DNA. This may constitute a mechanism of silver genotoxicity.^37^ Despite the ubiquitous presence of cysteine thiols within Zn(II) binding sites,^38,39^ the interaction of Ag(I) with zinc proteome remains largely unexplored. The interprotein zinc sites are particularly interesting, as they achieve highly distinctive properties upon the Zn(II) binding.^39−41^ Their formation, stability, and reactivity are driven by multiple factors, such as cellular or extracellular Zn(II) and protein subunit concentration, local redox environment, pH, and the presence of competitive ligands.^40^ The biological, physicochemical, and structural properties of the Zn(II)-dependent assembly of the Rad50 zinc hook complex have been investigated very thoroughly.^42−48^ Rad50 is a member of the Mre11–Rad50–Nbs1 (MRN) complex orchestrating the DNA double-strand break (DSB) repair.^42,43^ The functional Rad50 homodimer is stabilized by a tetrahedral Zn(II)–thiolate complex established by the two CXXC motifs. This motif is highly conserved in Rad50 homologs and was found across all forms of life, including bacteria, higher eukaryotes, and even viruses (Figure S1). In contrast to the classical zinc finger domain (ββα), where Zn(II) is bound by four donors (Cys_2_His_2_) from this domain, in the hook domain, each pair of Cys residues is donated by two separate Rad50 protomers. They are hitched together by a short β-hairpin motif and stabilized further by a set of interactions derived from the coiled-coil fragments (Figure 1).^43,44^ To ensure the activity of the whole MRN complex, the Rad50 zinc hook has to be hyperstable.^45,46^ Nevertheless, in this case, the hyperstability is not associated with selectivity, and the Zn(II) ion could be easily displaced by Cd(II) or Hg(II), yielding complexes with altered structure and enhanced stability and making Rad50 a potential target in Cd(II)- or Hg(II)-induced genotoxicity.^47,48^

The present work investigates the influence of Ag(I) ions on the Rad50 hook domain dimerization and its structure. For this purpose, we examined the Ag(I) interaction with two length-differentiated peptide models of the *Pyrococcus furiosus* hook domain, which is the best-described hook domain model so far. The peptide models were previously characterized with Zn(II) and toxic metals Cd(II) and Hg(II) (Hk14 and Hk45, Figure 1). Hk14 is a minimal model (violet in Figure 1) of Zn(II)-induced β-hairpin, while Hk45 is a full domain model (green + violet) covering the β-hairpin region and the adjacent coiled-coil fragments. These two constructs allowed us to determine the Ag(I) coordination properties of the metal binding center and correlate them with the structural stability of the hook domain fold. We used spectrophotometry, circular dichroism (CD), calorimetry, and mass spectrometry to demonstrate that Ag(I) can destabilize the Rad50 hook domain through Zn(II) displacement and disruption of the domain fold. This is the first report of Ag(I)-related destruction of the interprotein metal binding domain composed of two CXXC motifs. This result is likely to be generalized over other Zn(II)-based protein complexes, setting silver as a likely genotoxic agent with a broad range of deleterious effects.

## Experimental Methods

### Peptide Synthesis

The investigated Rad50 hook peptides were synthesized via solid-phase synthesis on TentaGel S Ram resin (substitution 0.22 mmol/g) using Fmoc-strategy^49^ and Liberty1 Microwave Peptide Synthesizer (CEM). The reagent excess, cleavage, and purification were performed as previously described.^45^ Briefly, the synthesized peptides were N-terminally acetylated using acetic anhydride and cleaved from the resin with a mixture of TFA/anisole/thioanisole/EDT/TIPS (88/2/3/5/2 v/v/v/v/v) over a period of 2.5 h followed by precipitation in cold (−80 °C) diethyl ether. The crude peptide was collected by centrifugation, dried, and purified via RP-HPLC (Dionex Ultimate 3000) using Phenomenex C18 columns and a linear gradient of ACN/water with 0.1% TFA from 1 to 90% of ACN over 25 min. The purified peptides were lyophilized, and their identity was confirmed by ESI-Q-TOF MS (Bruker Daltonik GmbH). The RP-HPLC chromatograms of purified peptides and the corresponding MS spectra are shown in Figures S2 and S3, respectively.

### Spectrophotometric Titration of Hk Peptides with Ag(I)

The UV–vis spectra of AgNO_3_-titrated Hk14 and Hk45 peptides were recorded at 25 °C under constant nitrogen flow in the wavelength range of 220–400 nm and a 1 cm pathlength quartz cuvette. Three accumulations were averaged using 200 nm·min^–1^ scanning speed and 2 nm bandwidth. Briefly, 25 μM of each Hk peptide in a degassed and chelexed 20 mM TES (100 mM NaF, pH 7.4) was titrated with small aliquots of concentrated Ag(I) solution from 0 to 4 molar Ag(I)/Hk ratio. The Hk solution was equilibrated 1 min after the addition of each portion of Ag(I). The AgNO_3_ titrations of Zn(Hk14)_2_ and Zn(Hk45)_2_ were performed analogously. All measurements were performed using a Jasco V-650 spectrophotometer with a Peltier heating/cooling system (Jasco).

### CD Titration of Hk Peptides with Ag(I)

The CD spectra of AgNO_3_-titrated Hk14 and Hk45 peptides were recorded at 25 °C under constant nitrogen flow in the wavelength range of 200–400 nm and a 2 mm pathlength quartz cuvette. Three accumulations were averaged using 200 nm min^–1^ scanning speed, 2 s digital integration time, and 2 nm bandwidth. Briefly, 25 μM of each Hk peptide in a degassed and chelexed 20 mM TES (100 mM NaF, pH 7.4) was titrated with small aliquots of concentrated AgNO_3_ solution from 0 to 4 molar Ag(I)/Hk ratio. The Hk solution was equilibrated 1 min after the addition of each portion of AgNO_3_. The AgNO_3_ titrations of Zn(Hk14)_2_ and Zn(Hk45)_2_ were performed analogously. All measurements were performed using a Jasco-1500 spectropolarimeter with a Peltier heating/cooling system (Jasco).

### Spectrophotometric PAR (4-(2-pyridylazo)resorcinol) Assay of Zn(II) to Ag(I) Swap

The Ag(I)-induced transfer of Zn(II) from either Zn(Hk14)_2_ or Zn(Hk45)_2_ to PAR was monitored spectrophotometrically at 25 °C under constant nitrogen flow and stirring (300 rpm) in a 1 cm quartz cuvette. The absorbance signal was monitored continuously using a kinetic mode at a fixed wavelength of 492 nm. The AgNO_3_ titrations of 10 μM Zn(Hk)_2_ complexes were performed in a degassed and chelexed 20 mM TES buffer (100 mM NaF, pH 7.4) in the presence of 100 μM PAR from 0 to 20 μM of added AgNO_3_. The Hk solution was equilibrated after each portion of added Ag(I) until the appearing curve, resulting from the Zn(II) to PAR transfer, reached a plateau. Finally, the molar concentration of the released Zn(II) was calculated using the effective molar absorption coefficient of the Zn(PAR)_2_ complex at pH 7.4, which is 71 150 M^–1^·cm^–1^.^50^ The measurements were performed using a Jasco V-650 spectrophotometer with a Peltier heating/cooling system (Jasco).

### Size-Exclusion Analysis of Ag(I)–Hk Complexes

Size-exclusion chromatography analyses were performed at room temperature using an NGC Quest 10 Plus Chromatography System (Bio-Rad). Samples of Hk14 (or Zn(Hk14)_2_) and Hk45 (or Zn(Hk45)_2_) were prepared in 20 mM TES, 100 mM NaF, pH 7.4 and run at 0.8 mL/min flow rate in the same buffer using Superdex 75 Increase 10/300 GL and Superdex 30 Increase 10/300 GL columns, respectively. Each sample was prepared immediately before the run by adding different molar equivalents of AgNO_3_. The column was equilibrated with at least two column volumes of the running buffer and calibrated with a mix of standard proteins prior to each experiment.

### ESI-MS of Ag(I)–Hk Complexes

The (+)ESI-MS-monitored AgNO_3_ titration of Hk peptides was performed in a degassed 50 mM ammonium acetate solution pH 7.4. The 2 μM samples of Hk14 or Hk45 were mixed with 0–4.0 mol equiv of AgNO_3_, incubated for 1 min, and injected by a syringe pump (10 μL/min) into an ESI-Q-ToF mass spectrometer (Compact Q-TOF, Bruker Daltonik GmbH). The MS spectra were recorded over 1 min in the positive ion mode within the 300–3000 *m*/*z* range at a 1 Hz acquisition rate. The following parameters were optimized to preserve peptide–metal ion complexes: capillary voltage of 3500 V, end plate offset potential of 500 V, nebulizer gas (N_2_) pressure of 0.4 bar, drying gas (N_2_) flow rate of 4 L/min, and drying temperature of 180 °C. The AgNO_3_ titrations of Zn(Hk14)_2_ and Zn(Hk45)_2_ complexes were performed analogously using the above conditions and parameters. The obtained mass spectrometry data were processed and analyzed using Compass DataAnalysis software (Version 5.1, Bruker Daltonik GmbH).

### Isothermal Titration Calorimetry (ITC)

The binding of Ag(I) to Hk14 and Zn(Hk14)_2_ was monitored with a Nano-ITC calorimeter (TA Instruments) at 25 °C in a Hastelloy cell of active volume of 1 mL in an overflow mode. All experiments were performed in 50 mM HEPES buffer, containing 100 mM NaF to establish the ionic strength at pH 7.4 under an argon atmosphere.^46^ In normal-mode experiments, the titrant (AgNO_3_) concentration was 6.03 mM and the titrate (Hk14 or Zn(Hk14)_2_) concentration was 150 μM. In the reverse mode, the titrant (Hk14 or Zn(Hk14)_2_) and titrate (AgNO_3_) were 1.3 mM and 36.2 μM, respectively. All titrant solutions were prepared freshly just before the measurement. After the initial temperature equilibration, a titrant solution was injected in a stepwise manner in 3 μL aliquots into the cell at 350 s intervals with stirring at 250 rpm. The control experiment, to determine the heat of titrant dilutions, was performed in the case of reverse-mode titration. Then, the net reaction heat was obtained by subtraction of the dilution heat from the corresponding total heat of injections. In the normal mode, the heat of titrant dilution was modeled from the final part of the isotherm. The data were analyzed in NanoAnalyze (version 3.12, TA Instruments), where the baseline and control heat subtractions were performed. Such preprocessed data were fitted to a one-site or multiple-site model supplied with the software. The error analysis was carried out by a Monte-Carlo approach with 1000 trials and a 0.95 level of confidence.

## Results and Discussion

Previous studies on the central fragments of the Rad50 protein from *P. furiosus* showed a strong relationship between the amino acid sequence, the tertiary/quaternary zinc hook domain structure, and the Zn(II) complex stability.^39,45,46^ The 14 amino acid long central fragment (Hk14, Figure 1) was found to form a highly structured minimal zinc hook domain upon the Zn(II) coordination. The 45 amino acid fragment (Hk45) was shown to form a full zinc hook domain, with all hydrophobic and electrostatic interactions. Therefore, herein, we used these two Rad50 fragments to study the principles governing the Rad50 hook domain interaction with Ag(I) ions, including their structural impact.

### UV–Vis and CD Study of Ag(I) Binding to Hk14 and Hk45 Metal-Free Peptides

**Figure 2:**
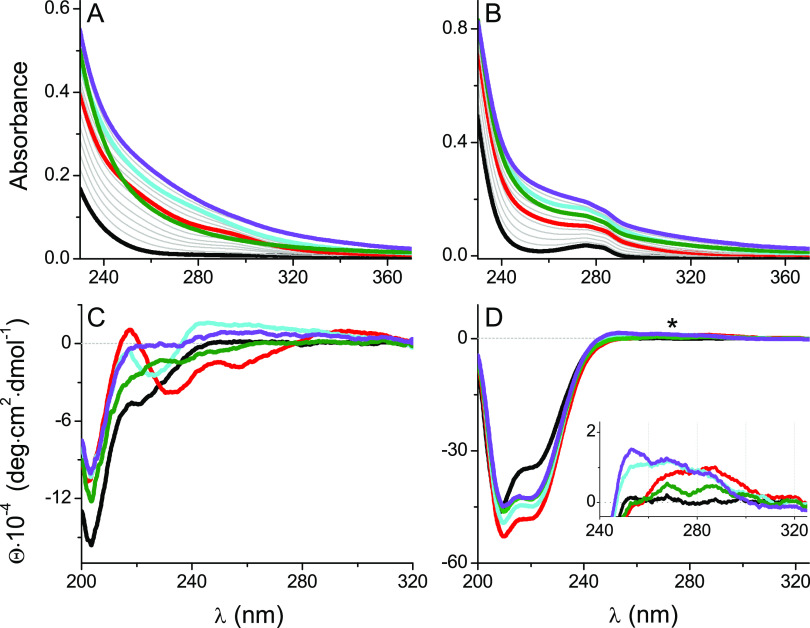
Spectrophotometric- (A, B) and CD- (C, D) monitored AgNO_3_ titrations of 25 μM Hk14 (left) and Hk45 (right) peptides in 20 mM TES, 100 mM NaF, pH 7.4. Black, red, cyan, green, and violet lines indicate 0, 1.0, 1.5, 2.0, and 3.0 Ag(I) mol equiv, respectively. Asterisk refers to signal changes presented in the inset of Figure 5C. The corresponding titration curves are presented in Figure 3.

UV–vis and CD-monitored AgNO_3_ titrations were performed to detect and characterize the Ag(I) binding to Hk14 and Hk45 peptides. The changes in the near-UV spectral range enabled us to follow the formation of Ag(I)–thiolate complexes, while the accompanying changes in the peptide secondary structure were followed at shorter wavelengths. Figure 2 presents the evolution of electronic absorption and CD spectra of Hk14 and Hk45 at pH 7.4 upon the additions of 0–3.0 mol equiv of Ag(I). Several ligand-to-metal charge transfer (LMCT) bands originating in Ag(I)–thiol bonds appeared in the 230–300 nm region similar to those reported for Ag(I) complexes of metallothionein (MT) and Ag(I)–ZF peptides.^36,51^ The alterations of band positions indicated the formation of various Ag(I) coordination species.

**Figure 3:**
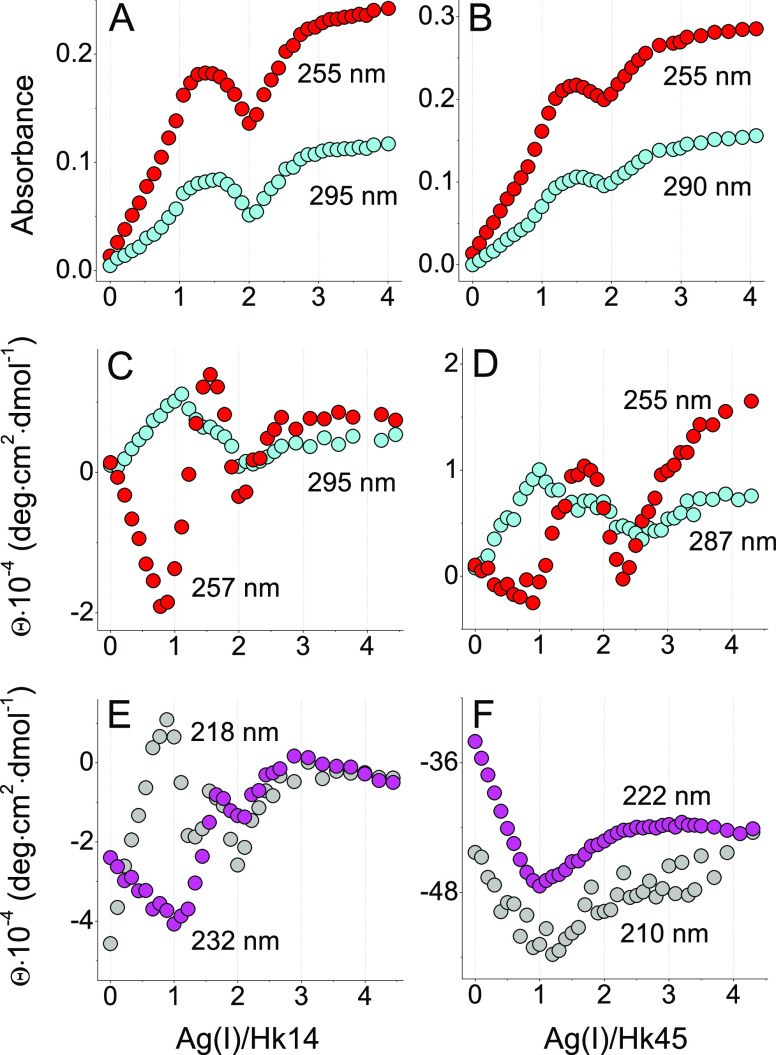
Spectrophotometric- (A, B) and CD- (C–F) monitored AgNO_3_ titrations of 25 μM Hk14 (left) and Hk45 (right) peptides in 20 mM TES, 100 mM NaF, pH 7.4 within 0–4.5 Ag(I) mol equiv. The corresponding absorbance and CD spectra are presented in Figure 2. The wavelengths in all graphs correspond to the region, where the signal change is most pronounced, thus providing the best signal-to-noise ratio.

Hk14 and Hk45 displayed similar Ag(I)-induced UV–vis spectra, although, for Hk45, the LMCT bands were somewhat obscured by the absorbance of the Tyr residue. The first added portions of Ag(I) induced the characteristic bands at 255–258 and 290–295 nm, which became saturated at ∼1.0 mol equiv of Ag(I), thus indicating the formation of either 1:1 or 2:2 Ag(I)-to-Hk complexes (see Figure 2A,B for the UV–vis spectra, Figure 2C,D for the CD spectra, Figure 3 for the corresponding titration curves, and Figure S5 presents the differential spectra, obtained by subtraction of the apo-Hk spectra). Upon further Ag(I) additions, the entire spectrum gained intensity, which reached a broad maximum within at ca. 1.5 Ag(I) mol equiv. Although the stoichiometries of Ag*_x_*(Hk)*_y_* complexes formed at this point are unclear, the two observed bands become less evident. The one at 295 nm is visibly blue-shifted, pointing to a change in the Ag(I) coordination environment. At the 2:1 Ag(I)-to-Hk molar ratio, the absorbance drops significantly and the plot minimum suggests a major complex rearrangement. It is even more evident when looking at the differential absorption spectra obtained by stepwise subtractions at 1, 1.5, 2, and 3 Ag(I) mol equiv (Figure S6). They show a decrease of the molar absorbance coefficient at 2 Ag(I) mol equiv. A further addition of AgNO_3_ caused the absorbance to increase again, but changes in the UV–vis spectra at 3.0 Ag(I) mol equiv and above are probably due to Hk aggregation initiated by such a high AgNO_3_ concentration. It could also be the result of the gradual oxidation of cysteine residues in the absence of the reducing agents that would hinder the interpretation of the data. However, this was ruled out by the DTNB assay, which demonstrated the stability of thiols during the time of the titration experiment (Figure S4). The characteristic bands at ∼250–260 and 280–295 nm observed throughout the spectroscopic titrations were previously noted in Ag(I) complexes with MT, Cys-bearing Cu(I)–chelators mimicking MT and Atox-1 sites, and in ZFs.^26,36,51^ Although they have been assigned as CT transitions of thiolate–Ag(I) bonds, they were not interpreted in terms of Ag(I) coordination geometry.

**Figure 4:**
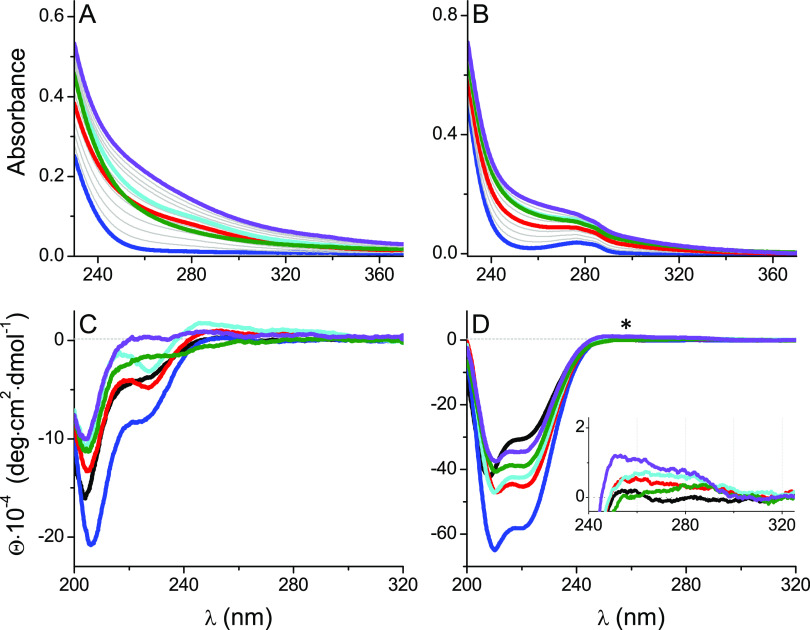
Spectrophotometric- (A, B) and CD- (C, D) monitored AgNO_3_ titrations of 25 μM Zn(Hk14)_2_ (left) and Zn(Hk45)_2_ (right) peptides in 20 mM TES, 100 mM NaF, pH 7.4. Black, blue, red, cyan, green, and violet lines indicate apo-Hk, Zn(Hk)_2_, 1.0, 1.5, 2.0, and 3.0 Ag(I) mol equiv, respectively. Asterisk refers to signal changes presented in the inset. The corresponding titration curves are presented in Figure 5.

The Ag(I)-induced changes in molar ellipticity of Hk peptides coincide with the ones observed in the UV–vis titrations. They display similar LMCT bands at the same Ag(I)-to-Hk stoichiometries (Figures 2C,D, 3C,D, and S5C,D). The first one appears after adding 1.0 Ag(I) mol equiv and corresponds to the formation of the 1:1 (or 2:2) Ag(I)-to-Hk species. The CD spectrum of Hk14 features a strong positive band at 218 nm, possibly coming from structural rearrangements, and two negative bands at 232 and 257 nm (Figures 2C and 3C,E). A broad positive band is also noticeable in the 280–320 nm range with a maximum at 295 nm. Further Ag(I) additions induced significant ellipticity changes, with turning points at ∼1.6, 2.0, and ∼3.0 Ag(I) mol equiv, above which no more characteristic bands were observed in the CD spectrum, like in the absorption spectra. Again, at 1.6 Ag(I) mol equiv, a weak positive band at 295 nm was shifted toward shorter wavelengths, which can be related to the formation of complexes with the larger nuclearity, favoring the diagonal AgS_2_ coordination mode. The negative bands at 232 and 257 nm disappeared above 1.6 Ag(I) mol equiv, indicating changes in S → Ag(I) band energies. Moreover, in the Hk45 variant, the addition of 1.0 Ag(I) mol equiv resulted in the partial α-helical peptide structurization, as evidenced by negative bands at 210 and 222 nm (Figures 2D and S5D). The turning point of the 222 nm CD signal (Figure 3F) illustrates the formation of a predominant AgHk45 (or Ag_2_(Hk45)_2_) species with high α-helical content, which suggests that at this ratio, Ag(I) is stabilizing some secondary structure in the Hk45 domain. The spectrum is, however, significantly different from that of the native Zn(II) complex (see Figure 4D).^46^ Additional Ag(I) ions caused a progressive α-helix loss (possibly by the coiled-coil structure disruption). These changes reach a plateau above 2.0 mol. equiv, whereas the LMCT changes are similar to those observed for Hk14 (Figure 3C–F). Unfortunately, the absence of theoretical analysis of S → Ag(I) in the literature precludes even tentative assignments of these bands to specific complex geometries.

The similar behavior of Hk14 and Hk45 in AgNO_3_ titrations suggests that the same types of Ag*_x_*(Hk)*_y_* complexes were formed regardless of the Hk peptide length. The gradual loss of secondary structure demonstrated by far-UV CD, accompanied by abrupt CD and absorption changes in the LMCT region, indicates that Ag*_x_*(Hk)*_y_* complexes of various stoichiometries were formed and that Ag(I) geometry evolved along the number of Ag(I) ions bound in a given complex. Nevertheless, the apparent formation of a mixed Ag*_x_*(Hk)*_y_* population makes it difficult to define the specific species.

### Ag(I) Binding to Zn(Hk14)_2_ and Zn(Hk45)_2_ Peptides: UV–Vis and CD Study

**Figure 5:**
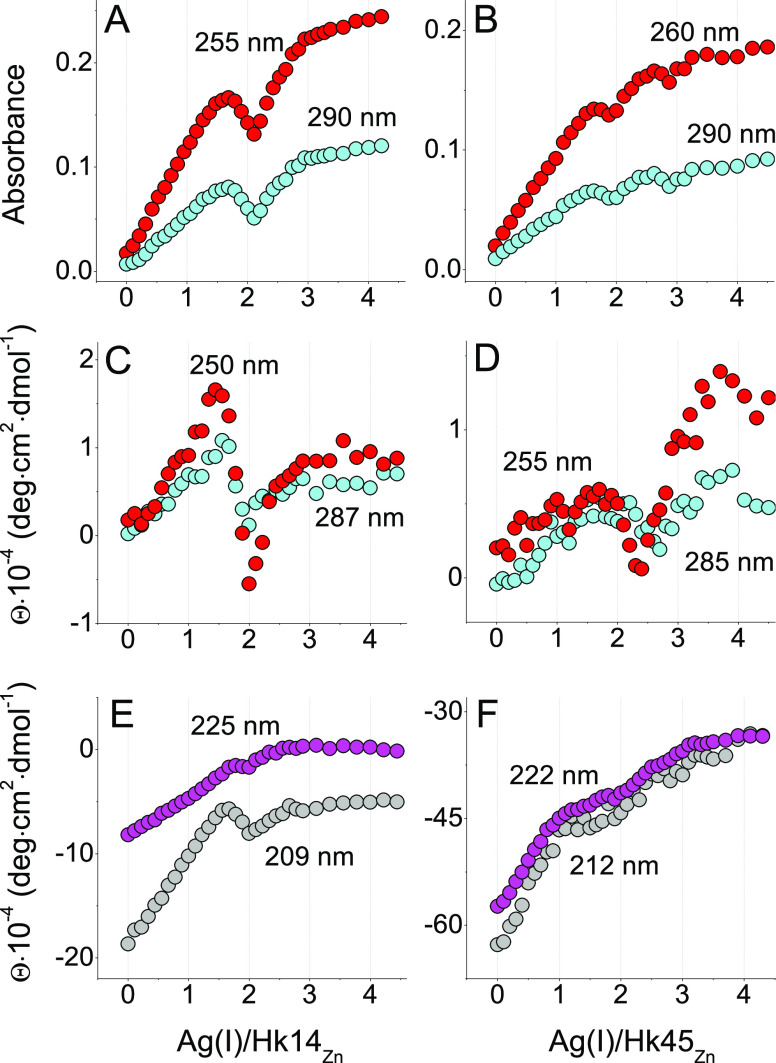
Spectrophotometric- (A, B) and CD- (C–F) monitored AgNO_3_ titrations of 25 μM Zn(Hk14)_2_ (left) and Zn(Hk45)_2_ (right) peptides in 20 mM TES, 100 mM NaF, pH 7.4 within 0–4.5 Ag(I) mol equiv. The *x*-axis represents the molar ratio of AgNO_3_ to the Hk monomer being in the Zn(II) complex. The corresponding absorbance and CD spectra are presented in Figure 4. The wavelengths in all graphs correspond to the region, where the signal change is most pronounced, thus providing the best signal-to-noise ratio.

To investigate whether the Zn(II) to Ag(I) swap observed in ZFs^36^ can also take place in the hook domain, its peptide models were first saturated with Zn(II) and then titrated with Ag(I) followed by UV–vis and CD spectroscopies. As with AgNO_3_ titration of the metal-free peptides, the absorbance spectra of Zn(Hk14)_2_ and Zn(Hk45)_2_ displayed Ag(I)-dependent changes between 230 and 300 nm, including the appearance of S → Ag(I) LMCT bands at ∼255 and 290 nm (Figures 4 and S7). Ag(I) can then bind to the cysteinyl ligands previously occupied by Zn(II), likely displacing it from its coordination site. When plotted against the Ag(I) mol equiv, the intensity of the two observed bands continuously increases up to ∼1.5, without a shoulder at 1.0 that was previously observed in titrated apo-peptides (Figure 5A,B). The characteristic broad maximum between 1 and 2 Ag(I) mol equiv was also absent. These initial differences in the UV range absorption trace for apo- and holo-Hk peptides suggest that the initial Zn(II) presence induced the preferable formation of specific Ag*_x_*(Hk)*_y_* complexes. This phenomenon can originate from the already preformed metal coordination center and the dimeric structure of Hk induced by Zn(II). Another possibility is that certain Ag*_x_*(Hk)*_y_* species are significantly less stable from the Zn(Hk14)_2_ complex^46^ and simply will not form in the presence of Zn(II). Upon further Ag(I) increase, both peptides displayed analogous changes to their zinc-free counterparts and after reaching minimum at 2.0 Ag(I) mol equiv, the signal continuously grew up to 3.0 mol equiv, over which no more new specific bands appeared that could be assigned to S → Ag(I).

CD-monitored AgNO_3_ titration of Zn(Hk)_2_ complexes partially clarified this issue (Figures 4C,D, 5C–F, and S7C,D). When looking at ellipticity changes within the CT range, the CD signal at 250–255 nm gradually increased up to ∼1.5 Ag(I) mol equiv. A minimum at 1.0 molar ratio did not appear (Figure 5C,D) and its lack coincided with negligible CT bands (compare the insets of Figures 2 and 4, red line) and the general loss of CD signal when compared to the corresponding changes observed for apo-Hk (Figure 4C,D, red line). This, again, indicates that the 1:1 complex either did not form or was not a predominant one during the Zn(II) substitution by Ag(I). Zn(Hk)_2_ is nevertheless significantly less stable than certain other Ag*_x_*(Hk)*_y_* complexes, which is indicated not only by the presence of S → Ag(I) CT bands but also by the continuous disappearance of minima characterizing β-fold and α-helical (coiled-coil) structure (Figure 5E,F). Indeed, the CD spectra characteristic for Zn(Hk14)_2_ and Zn(Hk45)_2_ disappeared along the Ag(I) additions, indicating the Ag(I)-induced structure disruption. This was somewhat expected due to a strong Ag(I) affinity toward “soft” sulfur donors. As a consequence, at a sufficiently high ratio, the added Ag(I) ions were able to disrupt the Zn(II)-induced native fold of not only the Zn(Hk14)_2_ β-hairpin complex but also the Zn(Hk45)_2_ dimer that is further stabilized by additional interactions within two monomers.

**Figure 6:**
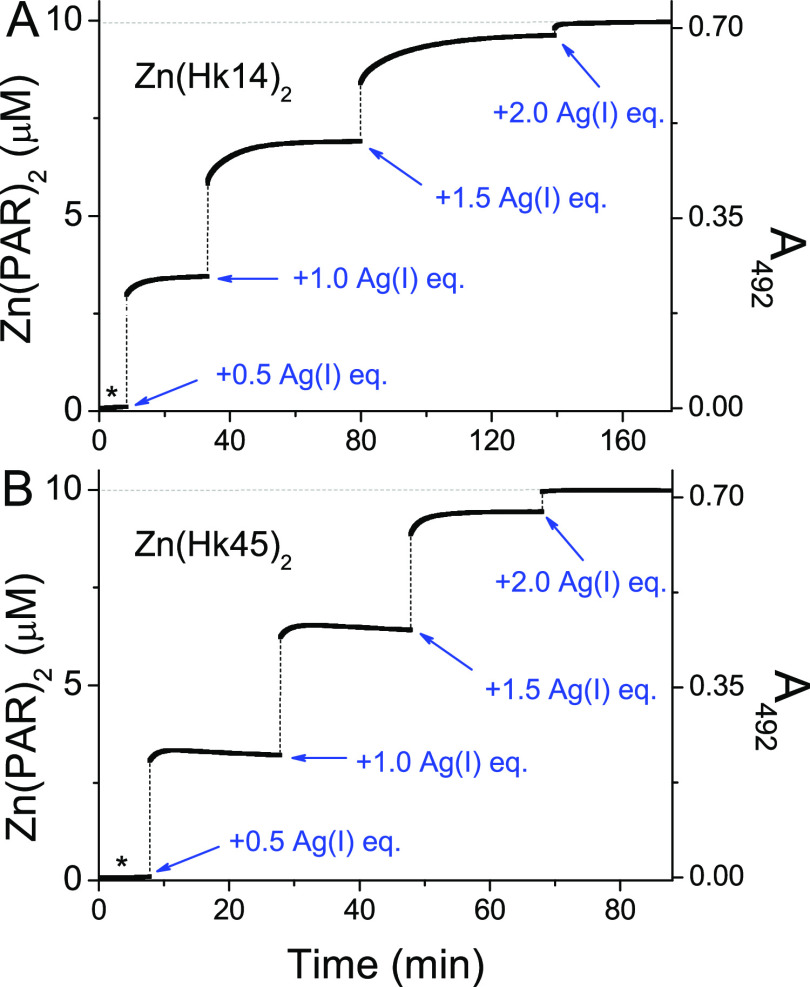
Zn(II) transfer from 10 μM Zn(Hk14)_2_ (A) and 10 μM Zn(Hk45)_2_ complex (B) to 100 μM PAR upon an addition of 0.5–2.0 mol equiv of Ag(I). The formation of the Zn(PAR)_2_ complex was monitored spectroscopically by measuring an increase in absorbance at 492 nm. AgNO_3_ titration was performed in 20 mM TES, 100 NaF, pH 7.4. Asterisk denotes the initial absorbance of Zn(Hk)_2_ before the addition of Ag(I).

To correlate the Ag(I) complexation process with Zn(II) release from Zn(Hk)_2_ complexes, the AgNO_3_ titration of holopeptides was repeated in the presence of Zn(II)-sensitive chromophore 4-(2-pyridylazo)resorcinol (PAR).^50^ The concomitant Zn(II) dissociation steps were monitored in a continuous mode at 492 nm to observe the formation of the Zn(PAR)_2_ complex (Figure 6). For both Hk14 and Hk45 peptides, the addition of 2.0 mol equiv of Ag(I) was sufficient to fully displace Zn(II) from its coordination site, as evidenced by the absorption increase. More than 90% of bound Zn(II) was released upon the addition of 1.5 Ag(I) mol equiv, which correlates with the maximum present in UV–vis and CD titration plots at 255 nm. Visibly slower swap kinetics for Hk14 than for the longer Hk45 peptide is noticed, which may arise from a higher fraction of various Ag*_x_*(Hk)*_y_* complexes that are formed upon the Ag(I) addition, which may require more time to form and rearrange due to a larger structuring.

### Oligomeric Ag(I)–Hk Complexes Demonstrated by Size-Exclusion Chromatography

**Figure 7:**
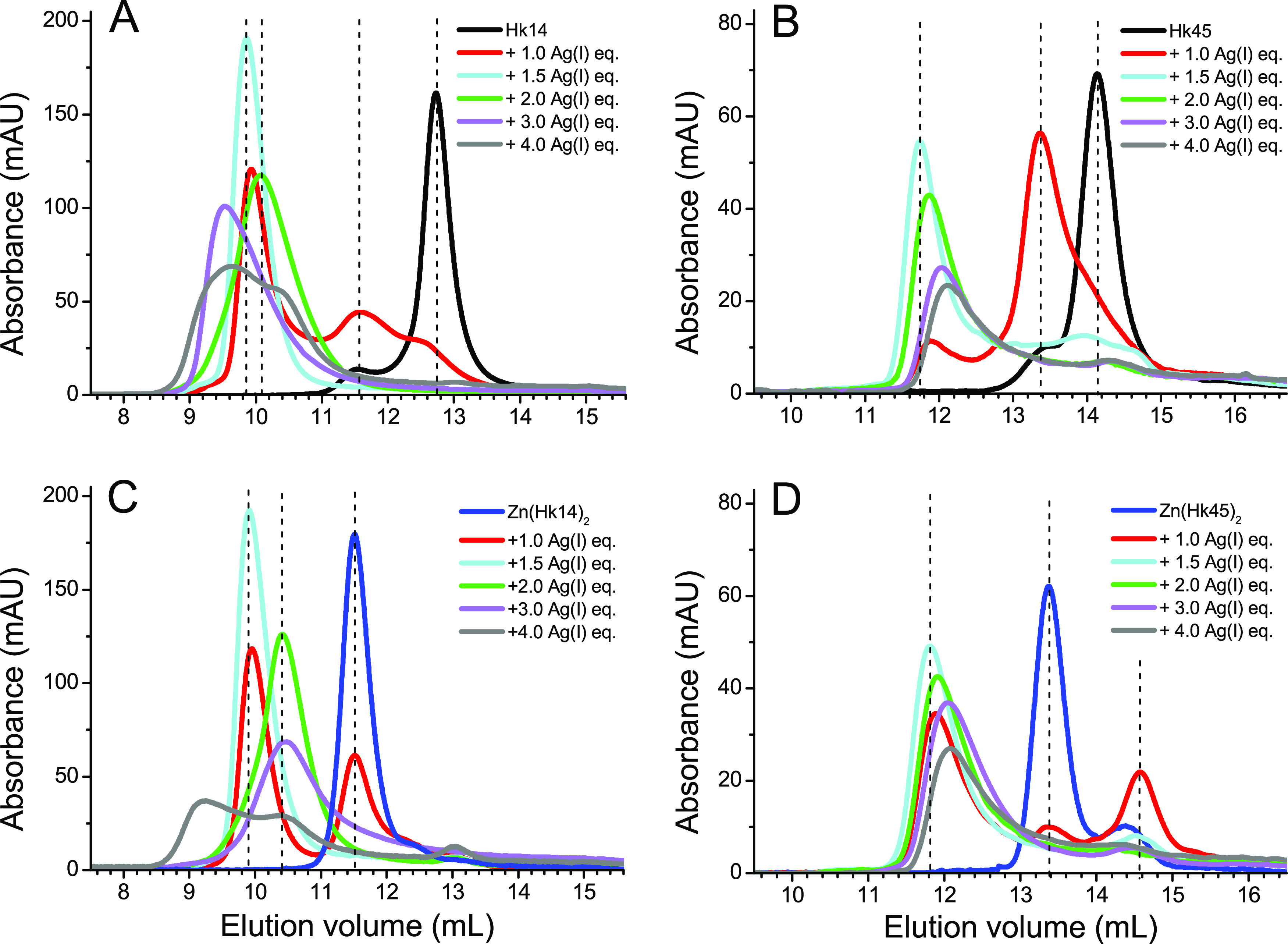
Size-exclusion chromatography analysis of Ag(I)–Hk14 and Ag(I)–Hk45 complexes. Elution profiles of metal-free Hk14 (A), Hk45 (B), Zn(Hk14)_2_ (C), and Zn(Hk45)_2_ (D) at different molar AgNO_3_ equivalents. Absorbance was measured at 220 nm. Dashed lines indicate the main peak signals.

The observations from spectroscopic experiments strongly suggest the formation of Hk complexes with different Ag(I)-to-Hk (or rather Ag(I)-to-Cys) stoichiometries. However, these data are not sufficient to tell monomeric from oligomeric Ag(I)/Hk complexes that may form in solution. We therefore performed size-exclusion analysis to assess the oligomerization state of the Ag(I)–Hk complexes depending on the Ag(I)-to-Cys molar ratio. Samples of Hk14 and Hk45 peptides either in apo- or Zn(II)-loaded forms were briefly incubated with different mol equiv of Ag(I) and run on SEC at room temperature. Figure 7A shows that the addition of 1.0 Ag(I) equivalent to Hk14 resulted in two signals markedly shifted from the apo-Hk14 monomer peak, a weak one at ∼11.5 min and the intense one at ∼10 min. The former one corresponds to the retention time of a dimeric Zn(Hk14)_2_, thus indicating the formation of Ag_2_Hk_2_. The latter one comes from the higher oligomeric complex. Lastly, a small intensity peak at 12.6 min points to the formation of AgHk species. These outcomes stay in agreement with the UV–vis and CD results (Figure 3A,C,E), where the titration curves revealed the formation of AgHk complexes. Especially, the CD data shows clearly that the AgHk—or rather—Ag_2_Hk_2_ dimer is formed, as it gains a certain level of structurization. The addition of another 0.5 Ag(I) equivalent caused the weaker signals to disappear, while the oligomer peak became dominant. This again corresponds to the maxima observed in spectroscopic titration curves at ca. 1.5 ratio. The addition of further Ag(I) equivalents weakened and shifted the peak slightly to ∼10.2 min. At this ratio, at which the minimum of the signal was observed in both UV–vis and CD titrations, the oligomeric complex likely rearranges into different species, in which either the oligomerization state has changed or the number of Ag(I) bound in the cysteine-rich core altered. Further addition of AgNO_3_ altered the overall signal shape and shifted it to ∼9.5 min, pointing to the formation of larger aggregates, as expected from UV–vis observations. The elution profiles obtained for Ag(I)-titrated Zn(Hk)_2_ were quite similar to that of the apo-peptide. The major peak at ∼10 min. (Figure 7C, cyan) likely corresponds to the maximum observed in the UV–vis and CD titration curves at ∼1.5 Ag(I)/Hk14 ratio (Figure 5A,C,E). An almost linear increase of the intensity of the UV–vis and CD bands up to this value indicates that this complex is preferably formed from the already preformed and structurally organized Zn(Hk14)_2_. It further agrees with the PAR assay results, where the addition of slightly more than 1.5 mol equiv of Ag(I) is sufficient to displace Zn(II) from its binding site (Figure 6). Again, at 2.0 mol equiv of Ag(I), the oligomeric complex rearranges into the complex with 2:1 Ag(I)-to-Hk stoichiometry (as indicated by the marked shift in the retention time) that transforms into higher aggregates upon further increase of Ag(I) concentration. The AgNO_3_ titration profiles of metal-free and Zn(II)-loaded Hk45 were similar to the ones observed for Hk14 and Zn(Hk14)_2_ (Figure 7B,D), although the formation of Ag_2_(Hk45)_2_ is much more pronounced than for Hk14, likely due to structurally determined arrangement of Cys residues of the longer polypeptide chain. For apo-Hk45, an addition of 1.0 Ag(I) mol equiv resulted in the major peak at 13.4 min, with the relative intensity much higher compared to the signal at 11.5 min for Hk14 at the same metal-to-peptide ratio (Figure 7B). The presence of AgHk45 (or partially structured Ag_2_(Hk45)_2_) was observed in UV–vis and CD titrations (Figure 3B,D,F). At 1.5 Ag(I) mol equiv, the signal from the larger complex increased, although, in contrast to the shorter Hk14 model, some signals corresponding to the dimeric and monomeric forms were still present. At 2.0 Ag(I) mol equiv, the main peak shifted, as for Hk14, indicating analogous reorganization of the complex. A similar trend was observed for Zn(Hk45)_2_ (Figure 7D), where the addition of 1.0 Ag(I) mol equiv disrupted the Zn(II)-bridged dimer into a monomer and higher oligomeric complex. Such disruption of Zn(Hk45)_2_ also demonstrates that the direct displacement of Zn(II) by the two Ag(I) ions in its coordination site would not preserve the proper structure of the hook dimer (see also Figures 4D and 5F).

It should be underlined that SEC is not fully quantitative because it separates particular species with respect to their hydrodynamic radii (i.e., size and shape), breaking the state of equilibrium. However, it is very convenient for examining various species formation or their transformation. SEC profiles of AgNO_3_-titrated Hk14 and H45 show that at the very first stage of Ag(I) complexation, a species with possible 2:2 stoichiometry is formed, which elutes with the same time as the Zn(Hk)_2_ species. Based on SEC profiles, this species turns at the 1.5 Ag(I)-to-Hk molar ratio into a species, which gives the highest signal for Hk14 and Hk45, respectively. This peak likely corresponds to higher Ag(I)-nuclearity. At a Ag(I)/Hk ratio of 2:1, the complex rearranges into another oligomeric form (and likely with a different number of bound Ag(I) ions), which either turns to higher aggregates (for Hk14) or starts to tail (for Hk45) along with further addition of AgNO_3_. One clear conclusion from SEC analysis is the subsequent Ag(I)-induced oligomerization of both peptides regardless of the original form.

### Identification of Ag(I)–Hk Complexes by ESI-MS

**Figure 8:**
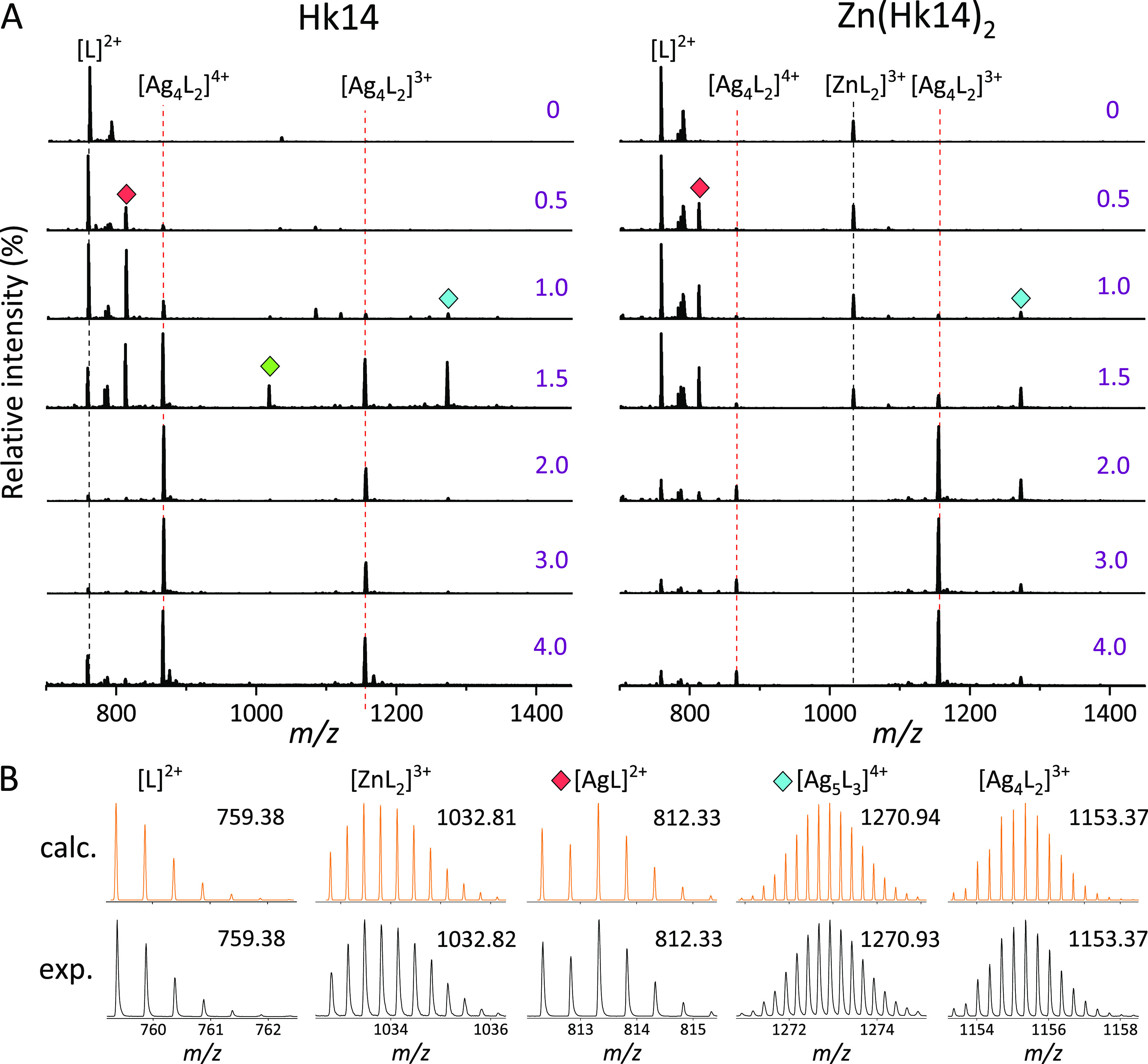
(+)ESI-MS-monitored AgNO_3_ titration of apo-Hk14 and Zn(Hk14)_2_ complexes. Mass spectra of 2 μM peptide solutions were recorded for 0–4.0 mol equiv of Ag(I) in 50 mM ammonium acetate, pH 7.4. (A) Red, green, and cyan labels denote [AgL]^2+^, [Ag_5_L_3_]^5+^, and [Ag_5_L_3_]^4+^ species, respectively. Dashed lines indicate substrates (black) and the final (red) AgNO_3_ titration product. (B) Comparison of the calculated and experimental isotopic patterns of the observed metal complexes.

Solution studies of Ag(I) binding to metal-free and Zn(II)-saturated Hk peptides were followed by electrospray ionization mass spectrometry (ESI-MS) analysis to identify the resulting Ag*_x_*(Hk)*_y_* products. ESI-MS preserves the noncovalent metal–protein interactions to some extent, especially those with large enthalpic components^52^ (e.g., soft metals with soft ligands). Hence, the specific Ag*_x_*(Hk)*_y_* complexes present in solution are likely to remain intact under gas-phase conditions. Apo-Hk peptides were initially dissolved in ammonium acetate pH 7.4 (50 mM salt concentration was used to minimize ESI-induced pH drop) and then either incubated with 0.5 Zn(II) mol equiv or directly saturated with increasing Ag(I) amounts, followed by a short incubation prior to the MS injection. Figure 8 presents the MS spectra obtained for Hk14, while the MS results for Hk45 and all of the assigned *m*/*z* signals are included in Figures S8 and S9 and Tables S1–S4, respectively.

Hk14 and Zn(Hk14)_2_ were measured first to assign the major *m*/*z* signals of the AgNO_3_ titration substrates (Figure 8A, black dashed lines). The initial addition of 0.5 Ag(I) mol equiv to either sample produced the Ag(Hk14) complex, appearing as a 2+ ion, accompanied by several low intensity peaks of other complexes, including Ag_2_(Hk14) and Ag_2_(Hk14)_2_ (Tables S1 and S2). In the presence of 1.0 Ag(I) mol equiv, the abundance of Ag(Hk14) significantly increased (the highest intensity), and new minor complexes were noticed, including the apo-Hk14 substrate. The predominance of Ag(Hk14), as well as the presence of Ag_2_(Hk14)_2_ signal, coincided with the turning point observed at 1.0 Ag(I) mol equiv in UV and CD titrations, confirming that 1:1 molar species were specifically formed in solution. At 1.5 Ag(I) mol equiv, a mixed population of Ag(Hk14), Ag_4_(Hk14)_2_, and Ag_5_(Hk14)_3_ complexes was detected for both Hk14 and Zn(Hk14)_2_, whereas signals from other species were vanishing. Such coexistence of various Ag(I) complex stoichiometries is consistent with spectroscopic and SEC results and further explains the noted difficulties in determining the Ag(I)-to-Hk binding stoichiometry. Ultimately, upon continuous Ag(I) additions, the two *m*/*z* signals corresponding to the Ag_4_(Hk14)_2_ complex dominated the mass spectra, especially at and above 2.0 mol equiv for both apo- and Zn(II)-bound peptides (Figure 8A, red dashed lines). This finding is consistent with the Zn(II) displacement experiment and demonstrates that at least at given gas-phase conditions, Ag_4_(Hk14)_2_ is the most stable complex that Ag(I) may form with Hk14.

The MS-monitored AgNO_3_ titration of Hk45 and Zn(Hk45)_2_ showed the formation of species with the same molar stoichiometries and the Ag_4_(Hk45)_2_ predominance, providing further evidence that Ag(I) readily replaces Zn(II) in the hook domain (Figures S8 and S9 and Tables S3 and S4). Table 1 summarizes major Ag*_x_*(Hk)*_y_* complexes detected by MS. The detection of the Ag_4_(Hk)_2_ complex is particularly interesting, as it is consistent with the result obtained for the Ag(I)-titrated CCCC ZF, for which the AgNO_3_ titration experiments revealed a stable Ag_4_ZF complex.^37^ The molecular dynamics and well-tempered metadynamics showed the formation of a Ag_4_(Cys)_4_ cluster in the ZF complex core. Analogously to the CCCC ZF, the two Hk monomers, each donating two cysteinyl residues, establish a tetrahedral coordination site for the Zn(II) ion. It is then possible, and consistent with the ESI-MS stoichiometry, that the Ag_4_(Cys)_4_ core can also be formed in the dimeric Hk system. It should be of course emphasized that ESI-MS is a qualitative rather than a quantitative method and that the appearing signals and their intensities corresponding to the metal–peptide complex present in the gas phase do not necessarily represent the presence and relative abundance of species existing in solution.^52,53^ Nevertheless, under the controlled pH and ionization conditions, the specific Ag(I)-to-Hk complexes that form in solution should be retained when transferred into the gas phase. The other potential risk to consider is the supermetallization^54^ of peptides during electrospray ionization. Yet, the observed 1:1 and 2:1 Ag(I)-to-Hk stoichiometries dominating in ESI-MS results converge with the stoichiometries observed in gel permeation and spectroscopic data.

**Table 1:** Summary of the Major Ag*_x_*(Hk14)*_y_* and Ag*_x_*(Hk45)*_y_* Complexes Observed in (+) ESI-MS Experiment^a^

Ag*_x_*L*_y_*	Hk14 (Da)	Hk45 (Da)	Ag(I) mol equiv
L	1516.7^b^	5161.8^b^	0
	1516.7^c^	5161.7^c^	
AgL	1622.6^b^	5267.6^b^	0.5–1.5
	1621.6^c^	5266.6^c^	
Ag_5_L_3_	5079.8^b^	16 017.6^b^	1.0–2.0
	5078.8^c^	16 013.6^c^	
Ag_4_L_2_	3457.1^b^	10 747.0^b^	1.5–4.0
	3457.1^c^	10 747.0^c^	

a Monoisotopic masses were derived from experimental isotopic patterns of the corresponding ion signals.

b Experimental *m*/*z* value.

c Calculated *m*/*z* value.

### Determination of Ag(I) Binding Affinity to Hk and Zn(Hk)_2_ by ITC

**Figure 9:**
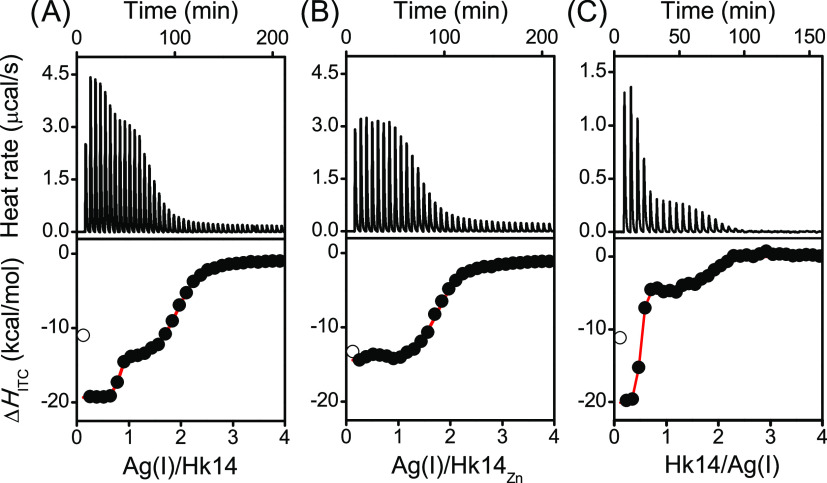
ITC analysis of Ag(I) binding to Hk14. ITC profile for titration of Ag(I) into Hk14 (A), Zn(Hk14)_2_ (B), and Hk14 into Ag(I) (C) in 20 mM HEPES, 100 mM NaF, pH 7.4. Top panels show the baseline-subtracted thermogram. The bottom panels represent the binding isotherm (see Table S5). The *x*-axis represents the molar ratio of AgNO_3_ to the Hk monomer being in the Zn(II) complex (A, B), and the molar ratio of Hk monomer to AgNO_3_ (C). Points not included in the fits were marked with white fill.

As shown recently, Zn(II) is easily displaced by Ag(I) from all ZF types, regardless of the number of Cys residues present in the zinc coordination site.^36,37^ Since Rad50 binds Zn(II) in a different stoichiometry than the single-molecule ZFs, a direct comparison of the respective affinities is not possible. It can be done, however, using the competitivity index (CI) method.^55^ The zinc hook domain from *P. furiosus* was shown to form complexes with subattomolar affinity, which in turn translates into CI that outperforms most of familiar ZFs, whose affinities do not exceed femtomolar values (10^–12^–10^–15^ M).^38,46^ Ag(I) titration to Hk14 and Hk45 (monitored by UV–vis, CD, and SEC) showed the formation of Ag(I)–Hk complexes of various stoichiometries, while the UV–vis-, CD-, and PAR-monitored titrations of Ag(I) to-Zn(Hk)_2_ complexes showed that the Zn(II)-to-Ag(I) substitution occurred spontaneously. The apparently full completion of the metal ion exchange for less than 2.0 molar excess of Ag(I) over the Zn(II) amount indicates that the Ag(I) affinity to the studied peptides must be at least 100-fold higher than that of Zn(II) (assuming that the noticeable amount of unreacted Zn(II) complex is less than 1% of the total signal). We applied ITC to estimate the stability difference of the hook complexes more precisely. The titration of Hk14 with Ag(I) ions showed two transitions at Ag(I)-to-Hk14 molar ratios 0.8 and 2.1. The latter, taken together with the UV–vis, CD, and MS data, strongly suggests the formation of the Ag(I)–Hk complex of 2:1 stoichiometry (Figure 9A and Table S5). The first step of complexation is a proton-linked process; thus, the observed Δ*H*_ITC_ is a sum of several effects: deprotonation of the Hk14 peptide upon metal complexation, protonation of buffer component, and the binding of Ag(I) to deprotonated thiol groups (thiolates). The second transition represents the end of Ag(I) binding to Hk14. The reverse Hk14-to-AgNO_3_ titration (Figure 9C) confirms this scenario. Interestingly, compared to the previously used methods, ITC does not show the formation of any other Ag(I)–Hk complexes, which may be due to the possibility that their formation is not accompanied by distinguishable changes in enthalpy. What is worth noting is the fact that AgNO_3_ addition to the preformed Zn(Hk14)_2_ complex yielded only one simultaneous step of Ag(I) complexation (at the inflection point of ∼2) and Zn(II) dissociation from the already deprotonated sulfur donors (Figure 9B). Direct Ag(I)-to-Hk14 and Hk14-to-Ag(I) titration experiments were fitted to multiple site model while Ag(I)-to-Zn(Hk14)_2_ titrations to independent site model (Table S5, control titrations Figure S10). Dissociation constants for the first cannot be considered because very high Ag(I)–Hk complex affinity is out of the effective range of the ITC method. *K*_d_ obtained from the fitting of Ag(I) titration to the preformed Zn(Hk14)_2_ complex is 4.1 μM and remains in the effective range of ITC. In fact, the obtained *K*_d_ should be considered here as an exchange constant (*K*_ex_),^50^ which allows estimating affinity of Ag(I)–Hk species by this value multiplying by *K*_12_ of Zn(Hk)_2_. The affinity of the Ag(I) complex is therefore ∼240 000-fold higher (low zeptomolar range) than the Zn(II) one taking into account known −log *K*_12_ = 19.9 for Zn(Hk14)_2_.^46^

### Rad50 Activity Disruption by Ag(I) Relies on Zn(II) Swap

**Figure 10:**
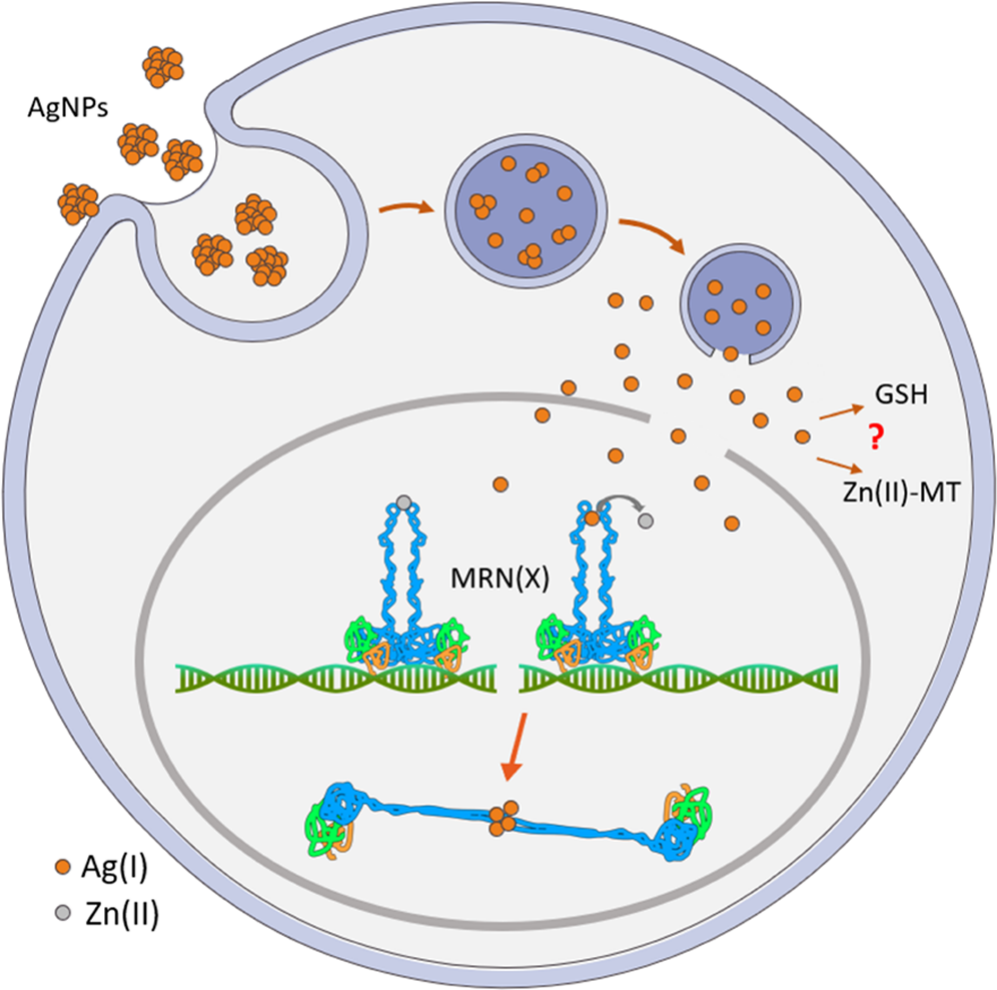
Proposed pathway of AgNP transfer to the cell, their dissolution and Ag(I) release in nuclear peripheries. Ag(I) ions are translocated to the nucleus and interact with Zn(II)–thiolate binding sites, such as Rad50 being a part of the MRN(X) complex. Zn(II)-to-Ag(I) swap alters the structure of the dimeric hook domain and generates dysfunctional MRN complexes. Graph has been prepared using Servier Medical Art https://smart.servier.com.

The AgNP uptake into the cell is an active process; apart from this, there is no clear consensus on the AgNP uptake pathway.^17^ Inside the cell, silver species coexist as Ag(0) that form AgNPs and as organothiol-Ag(I). The ratio of the species depends on various factors, such as duration of exposure, dose, and cell type. The process of Ag(I) leaching from AgNPs is conducted inside the endolysosomal vesicles; the decrease of pH and the accompanying chloride concentration cause the release of soluble AgCl*_x_*.^17^ Within the endoplasm, Ag(I) is present mostly as organothiol complexes, from which the exchange of Ag(I) to Cu(I) or Zn(II) from various metalloproteins can occur.^17,18^ Ag(I) ions can be translocated to the nucleus and interact with physiologically occurring Zn(II)–thiolate binding sites, i.e., zinc binding sites in ZFs in transcription factors, as well as in the intermolecular zinc binding site in Rad50 (Figure 10).^15,18^ The possible Rad50 activity disruption by Ag(I) may rely on Zn(II) swap and formation of Ag(I)–Hk species, which significantly affect the structure of the Rad50 hook. As has been established by other studies, even a small mutation in the Rad50 hook may lead to a huge biological impact, thus by analogy, the Rad50 mismetallation by Ag(I) may lead to impairment of at least some of the Rad50 functions or even the whole MRN complex.^44,56^

## Conclusions

Our study demonstrates that Ag(I) ions can directly replace Zn(II) not only in zinc finger domains but also in unique binding sites located on the interface between interacting proteins. Binding of Ag(I) to the Rad50 hook domain exhibited complicated speciation of the formed Ag(I)–Hk complexes, which likely coexisted in solution during the direct titration experiments. Complexes of various stoichiometries were found, among which Ag(I)–Hk species of 1:1 and 2:1 molar ratio were formed preferentially when a sufficient amount of Ag(I) ions was available.^36,37^ The stability of the latter one was at least 5 orders of magnitude higher than the high attomolar one of Zn(Hk)_2_. This indicates the Ag(I)-to-hook domain affinity is in the low zeptomolar range. The formation of the underlying Ag_4_(Cys)_4_ core results in the native structure disruption of the Zn(Hk)_2_ complex, which is highly important for the proper functioning of the MRN complex in DNA DSB repair. The observed association tendency of Ag(I)–Hk species also indicates that the disruption of Rad50 may occur due to its irreversible oligomerization. The presence of many Cys-rich binding sites in DNA and RNA processing proteins strongly suggests that the loss of their native function and structure can be the basis of silver genotoxicity.

## Supporting Information Available

Materials; RP-HPLC chromatograms of purified Hk14 and Hk45 peptides and the corresponding ESI-MS spectra; figures with spectra from UV–vis, CD, and ESI-MS experiments; tables with all the assigned *m*/*z* values in ESI-MS experiments; supplementary table with values calculated in the ITC experiment (PDF)
