# Supplementary material for: Zn(II) to Ag(I) Swap in Rad50 Zinc Hook Domain Leads to Interprotein Complex Disruption through the Formation of Highly Stable Agx(Cys)y Cores
Source: Inorg Chem. 2023 Mar 2;62(10):4076–87. doi: 10.1021/acs.inorgchem.2c03767 (PMC10015552; doi:10.1021/acs.inorgchem.2c03767)
Supplement: Supplementary file 1 — ic2c03767_si_001.pdf [file ic2c03767_si_001.pdf]

Supporting Information

Zn(II) to Ag(I) swap in Rad50 zinc hook domain leads to interprotein complex disruption through the formation of highly stable $Ag_x(Cys)_y$ cores

Olga Kerber,[†] Józef Tran,[†] Alicja Misiaszek,[†] Aleksandra Chorążewska,[†] Wojciech Bal[‡] and Artur Krężel[†*]

[†] *Department of Chemical Biology, Faculty of Biotechnology, University of Wrocław, Joliot-Curie 14a, 50-383 Wrocław, Poland*

[‡] *Institute of Biochemistry and Biophysics, Polish Academy of Sciences, Pawińskiego 5a, 02-106 Warsaw, Poland*

TABLE OF CONTENTS

Materials……………………………………………………………………………....S2
Figure S1. ……………………………………………………………………………S3
Figure S2……………………………………………………………………………...S4
Figure S3. …………………………………………………………………………… S5
Figure S4…………………………………………………………………………….. S6
Figure S5……………………………………………………………………………...S7
Figure S6……………………………………………………………………………...S8
Figure S7……………………………………………………………………………...S9
Figure S8……………………………………………………………………………S10
Figure S9…...………………………………………………………………………..S11
Figure S10...………..………………………………………………………………..S12
Table S1……………………………………………………………………………..S13
Table S2……………………………………………………………………………..S14
Table S3……………………………………………………………………………..S15
Table S4……………………………………………………………………………..S16
Table S5……………………………………………………………………………..S17
References…………………………………………………………..………………S18

Materials

The following reagents were purchased from Sigma-Aldrich: $ZnSO_4 \cdot 7H_2O$, $AgNO_3$, ammonium acetate, NaF, 4-(2-pyridylazo)resorcinol (PAR), 1,2-ethanedithiol (EDT), thioanisole, anisole, triisopropylsilane (TIPS). The metal-chelating resin Chelex 100 was acquired from Bio-Rad. 2-{[1,3-Dihydroxy-2-(hydroxymethyl)propan-2-yl]amino}ethane-1-sulfonic acid (TES), 4-(2-hydroxyethyl)-1 piperazineethanesulfonic acid (HEPES) were obtained from ROTH and BioShop, respectively. *N*,*N*-dimethylformamide (DMF) was purchased from VWR Chemicals. Acetonitrile (ACN) was acquired from Merck Millipore. Acetic anhydride, diethyl ether, dichloromethane (DCM) were purchased from Avantor Performance Materials Poland (Gliwice, Poland). 1-Methyl-2-pyrrolidinone (NMP), *N*,*N*,*N′*,*N′*-tetramethyl-*O*-(1*H*-benzotriazol-1-yl)uronium hexafluorophosphate (HBTU), trifluoroacetic acid (TFA), *N*,*N*-diisopropylethylamine (DIEA), piperidine, TentaGel S Ram and Fmoc-protected amino acids were obtained from Iris Biotech GmbH (Marktredwitz, Germany). The concentration of metal ion salt stock solutions was 0.05 M and was confirmed by a representative series of ICP-MS measurements. All pH buffers were treated with Chelex 100 resin to eliminate trace metal ion contamination.

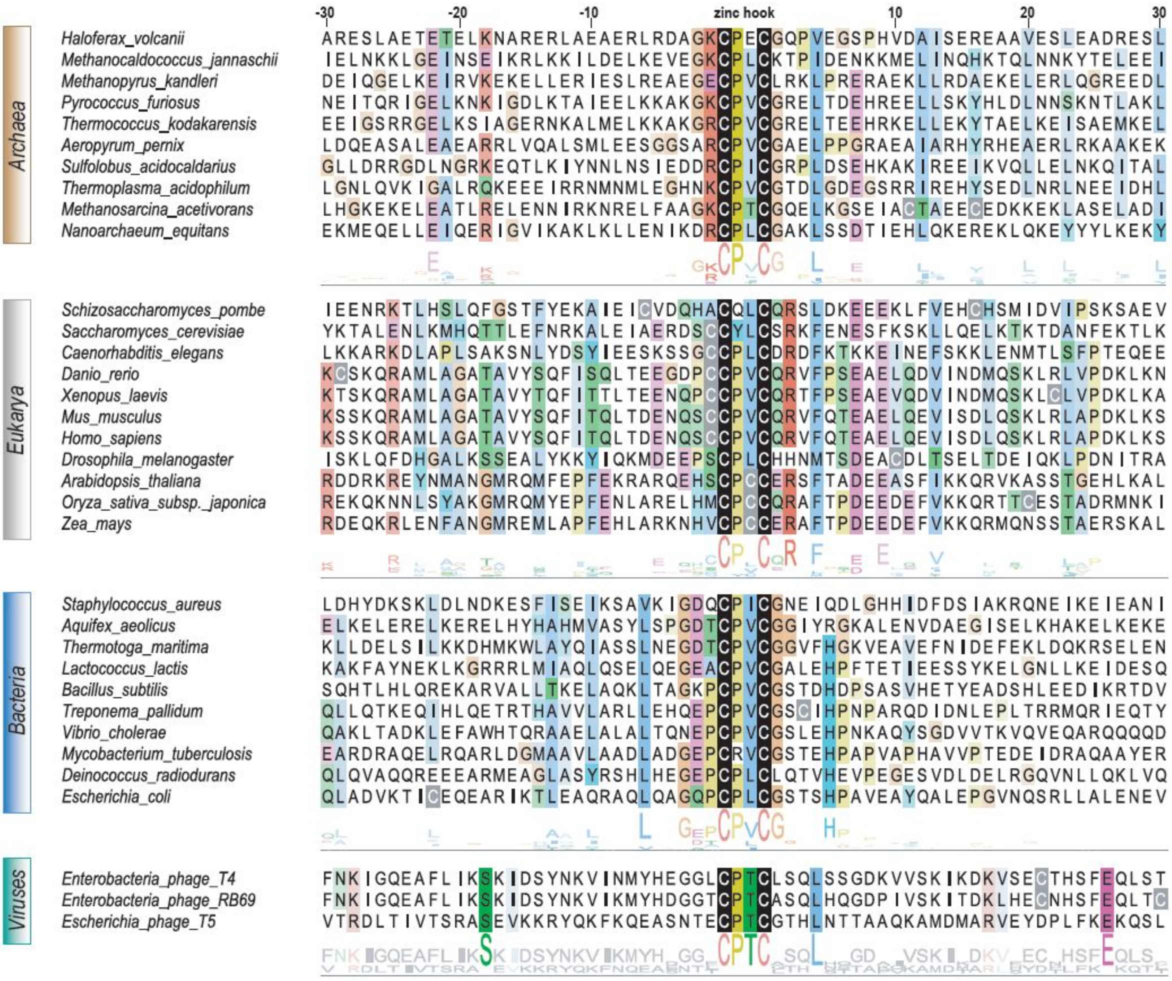

Figure S1. Protein sequence alignment of representative 64 amino-acid long Rad50 fragments covering the entire zinc hook domain with a stretch of coiled-coil on either side. The CXXC motif is positioned in the middle of the sequences. Sequence logos correspond directly to the shown sequences. Figure was adopted from Padjasek *et al.*[1]

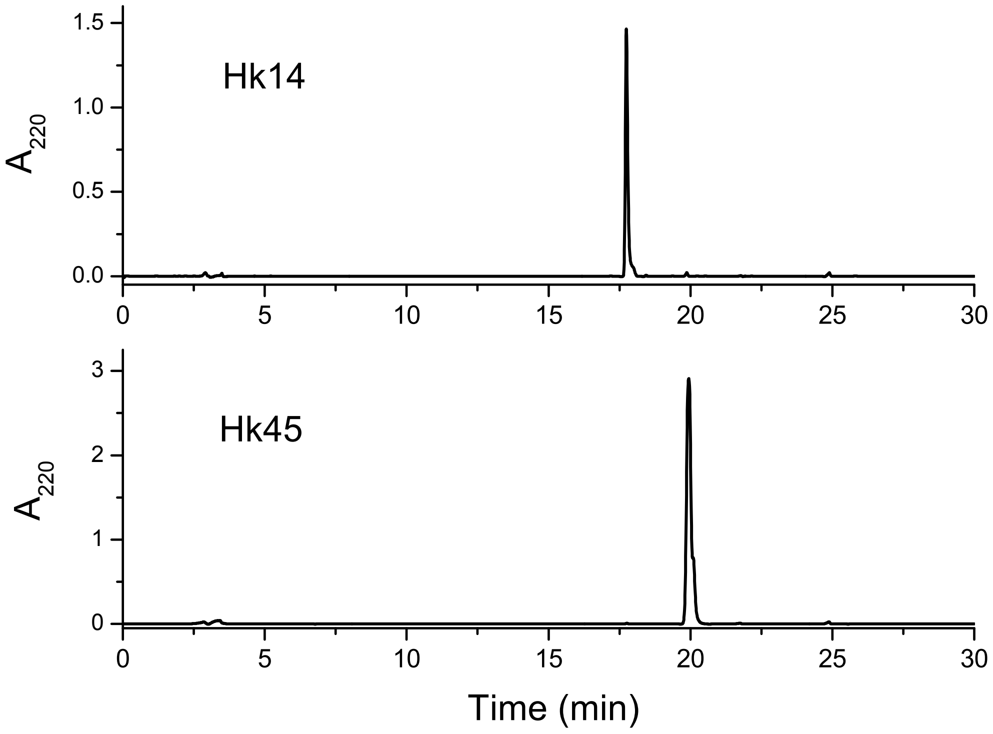

Figure S2. RP-HPLC chromatograms of synthetized Hk peptides purified on a Phenomenex Jupiter C18 column (250 mm × 4.6 mm, 5.0 µm). Top: Hk14, retention time 17.2 min. Bottom: Hk45, retention time 19.9 min. The parameters of the method were as follows: 0.1% TFA in water and 1% of ACN for the first 5 min, then linear gradient from 1% to 90% ACN/water with 0.1% TFA in 25 min, flow rate of 1.0 ml/min.

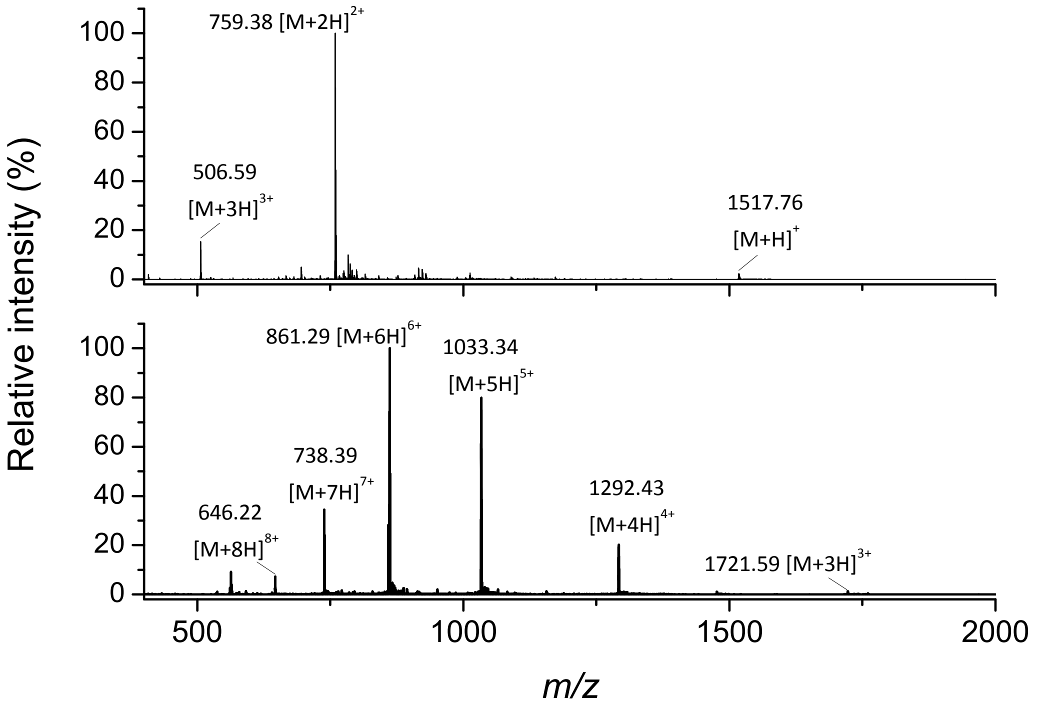

Figure S3. (+)ESI-MS spectra of synthetized Hk14 (top) and Hk45 (bottom) peptides recorded in 50/49/1 (v/v/v) MeOH/water/formic acid. The expected/calculated monoisotopic masses are 1516.738/1516.751 Da and 5161.691/5161.687 Da, respectively.

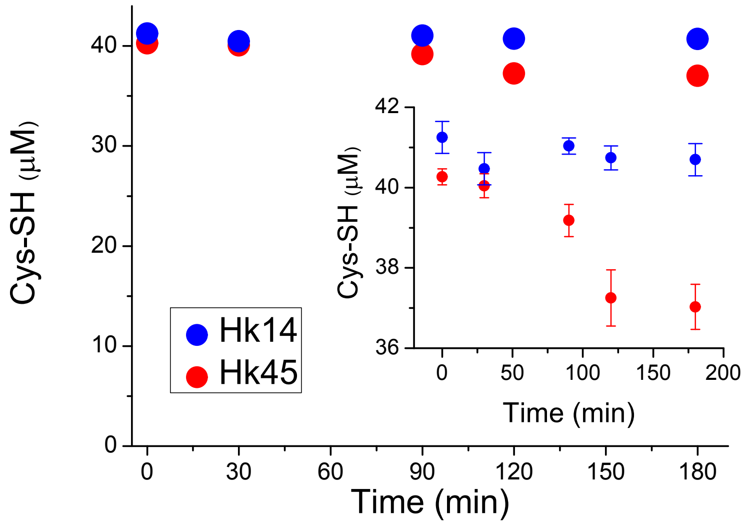

Figure S4. UV-vis-monitored oxidation of 20 μM Hk14 and Hk45 peptides using DTNB reagent. Samples containing metal-free Hk14 (blue) or Hk45 (red) peptide dissolved in 20 mM TES, 100 mM NaF, pH 7.4 were incubated for 0, 30, 90, 120, and 180 minutes. After incubation, DTNB was added to a final concentration of 1 mM, and the absorbance of the samples was measured at 412 nm. The obtained values were used to calculate the molar concentration of the reduced cysteinyl residues using the molar extinction coefficient of 14,150M^{-1} cm^{-1}.[2] The graph presents changes in the reduced Cys level *vs.* time through which the Hk remained in the buffered solution. Error bars are included in the inset.

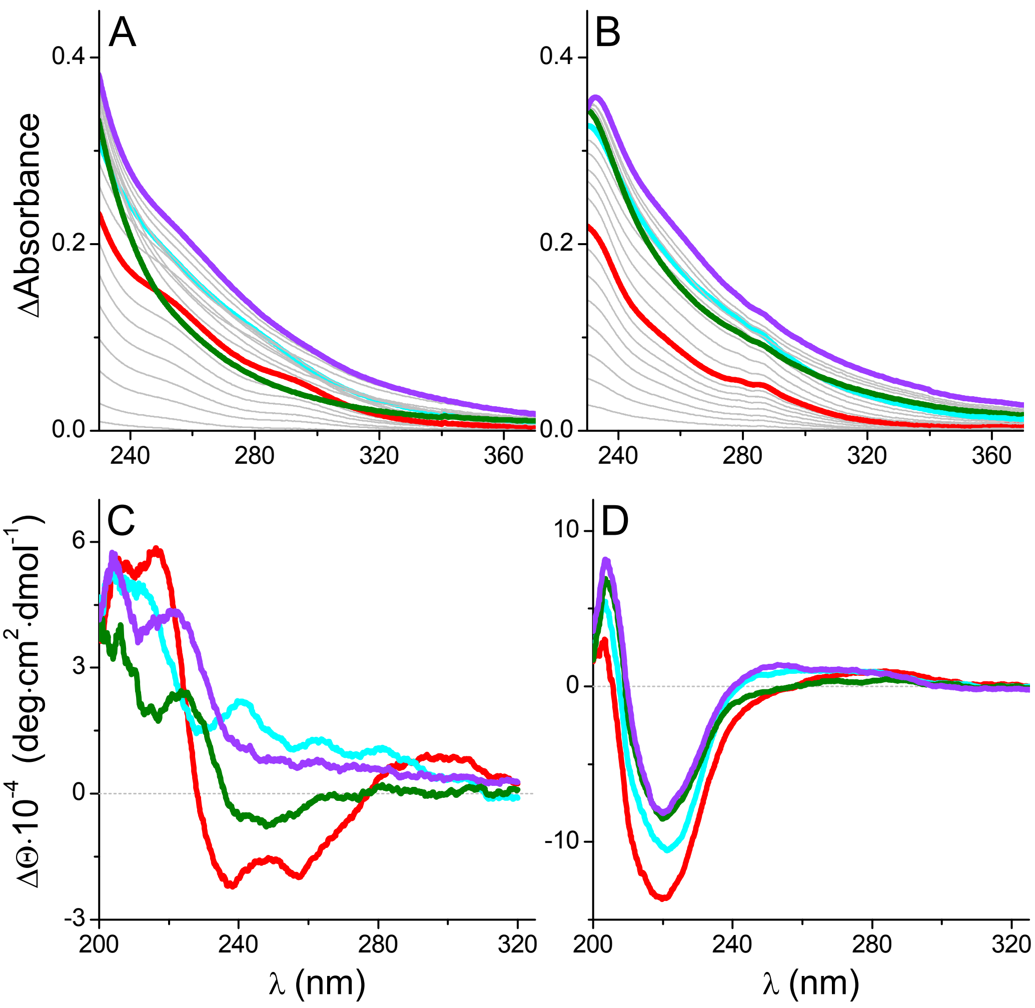

Figure S5. UV-vis (top) and CD (bottom) differential spectra of Ag(I)-titrated 25 μM Hk14 (A,C) and Hk45 (B,D) obtained by subtraction of apo Hk spectrum. All spectra were recorded in 20 mM TES, 100 mM NaF, pH 7.4. Red, cyan, green and violet lines indicate for 1.0, 1.5, 2.0 and 3.0 Ag(I) mol. eq., respectively.

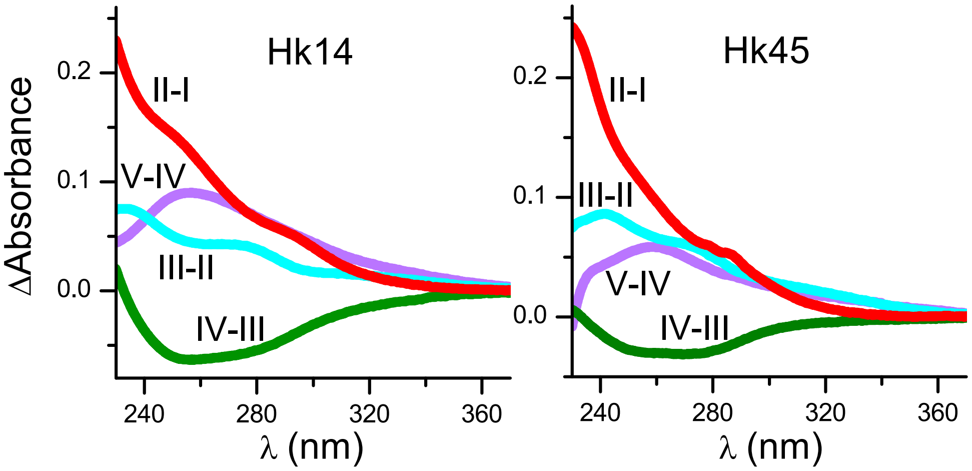

Figure S6. Increment difference absorption spectra corresponding to the spectrophotometric $AgNO_3$ titration of Hk14 and Hk45 showed in Figure 2. Red, cyan, green, and violet lines indicate for subtraction of spectrum II - spectrum I, III-II, IV-III, and V-IV, where I denotes 0, II – 1.0 and 0.9, III – 1.5 and 1.4, IV – 2.0 and 1.9, V – 3.0 Ag(I) mol. eq. for Hk14 and Hk45, respectively.

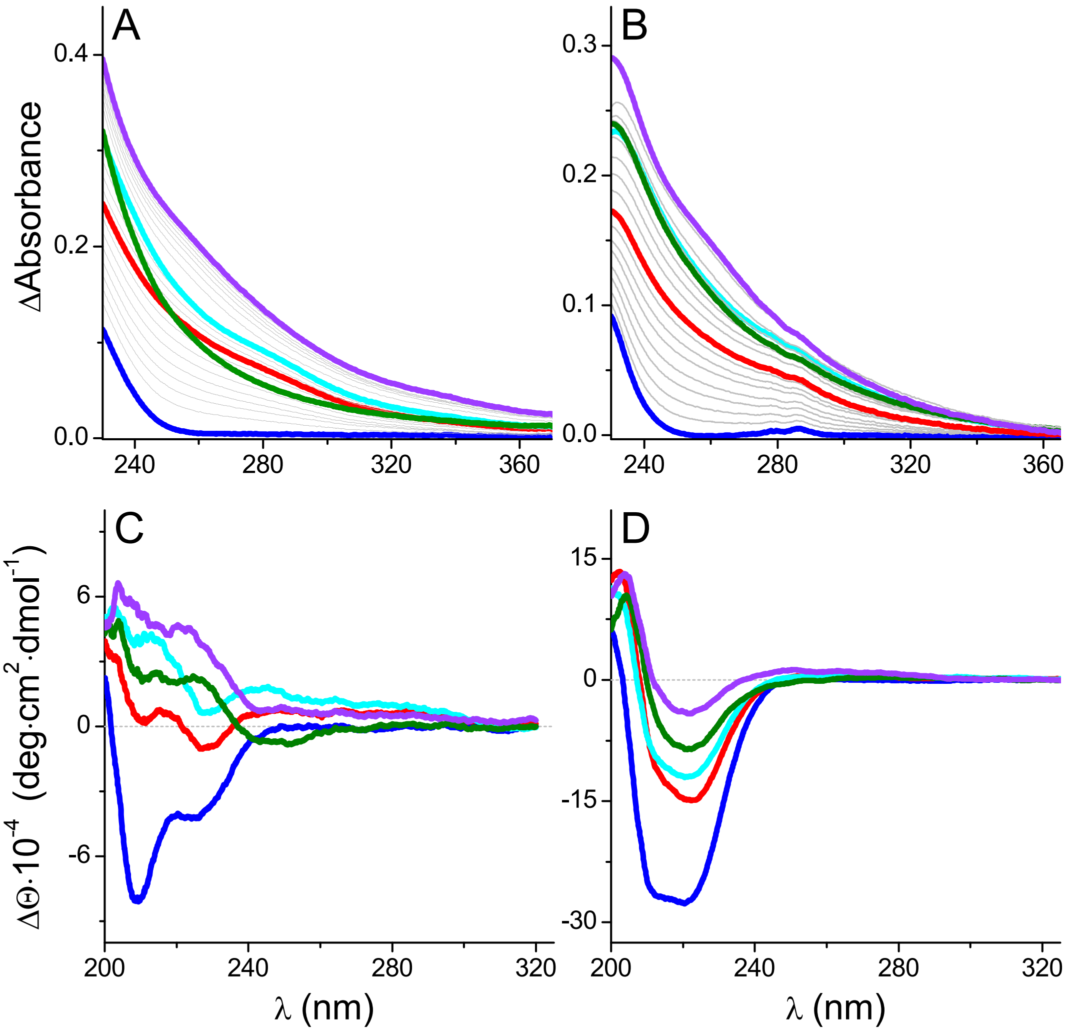

Figure S7. UV-vis (top) and CD (bottom) difference spectra of Ag(I)-titrated 25 μM $Zn(Hk14)_2$ (A,C) and $Zn(Hk45)_2$ (B,D) complexes obtained by subtraction of apo Hk spectrum. All spectra were recorded in 20 mM TES, 100 mM NaF, pH 7.4. Blue, red, cyan, green and violet lines indicate 0, 1.0, 1.5, 2.0, and 3.0 Ag(I) mol. eq., respectively.

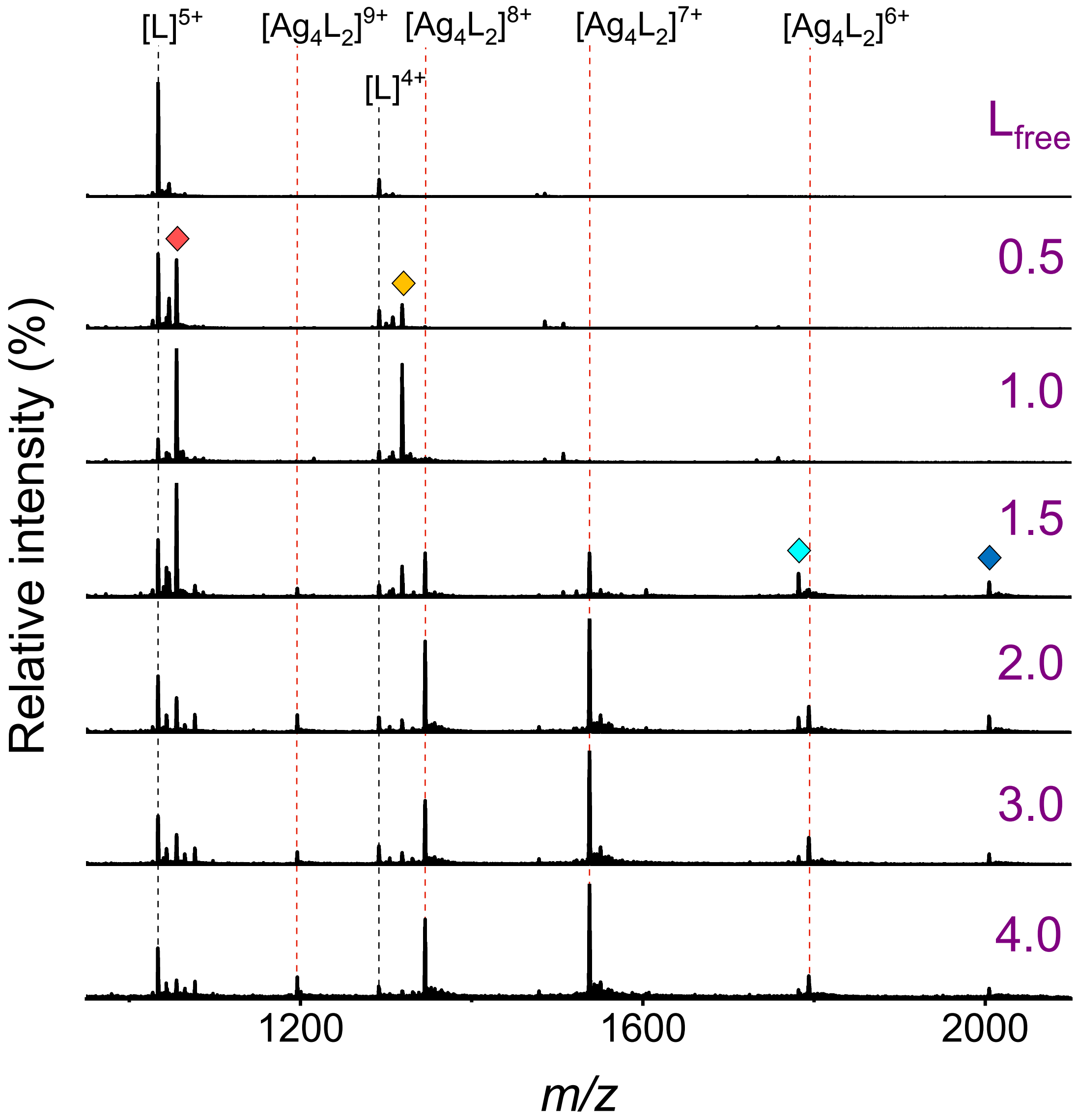

Figure S8. (+)ESI-MS-monitored Ag(I) binding to apo Hk45. Mass spectra of 2 µM peptide solutions were recorded for 0-4.0 mol. eq. of Ag(I) (violet color) in 50 mM ammonium acetate, pH 7.4. Black and red dashed lines represent L (Hk45 monomer) and Ag_4L_2 ions as the substrate and the final product of $AgNO_3$ titration, respectively. Red, orange, cyan, and blue labels denote $[AgL]^{5+}$, $[AgL]^{4+}$, $[Ag_5L_3]^{9+}$, and $[Ag_5L_3]^{8+}$ species, respectively. Other assigned m/z signals are presented in Table S3.

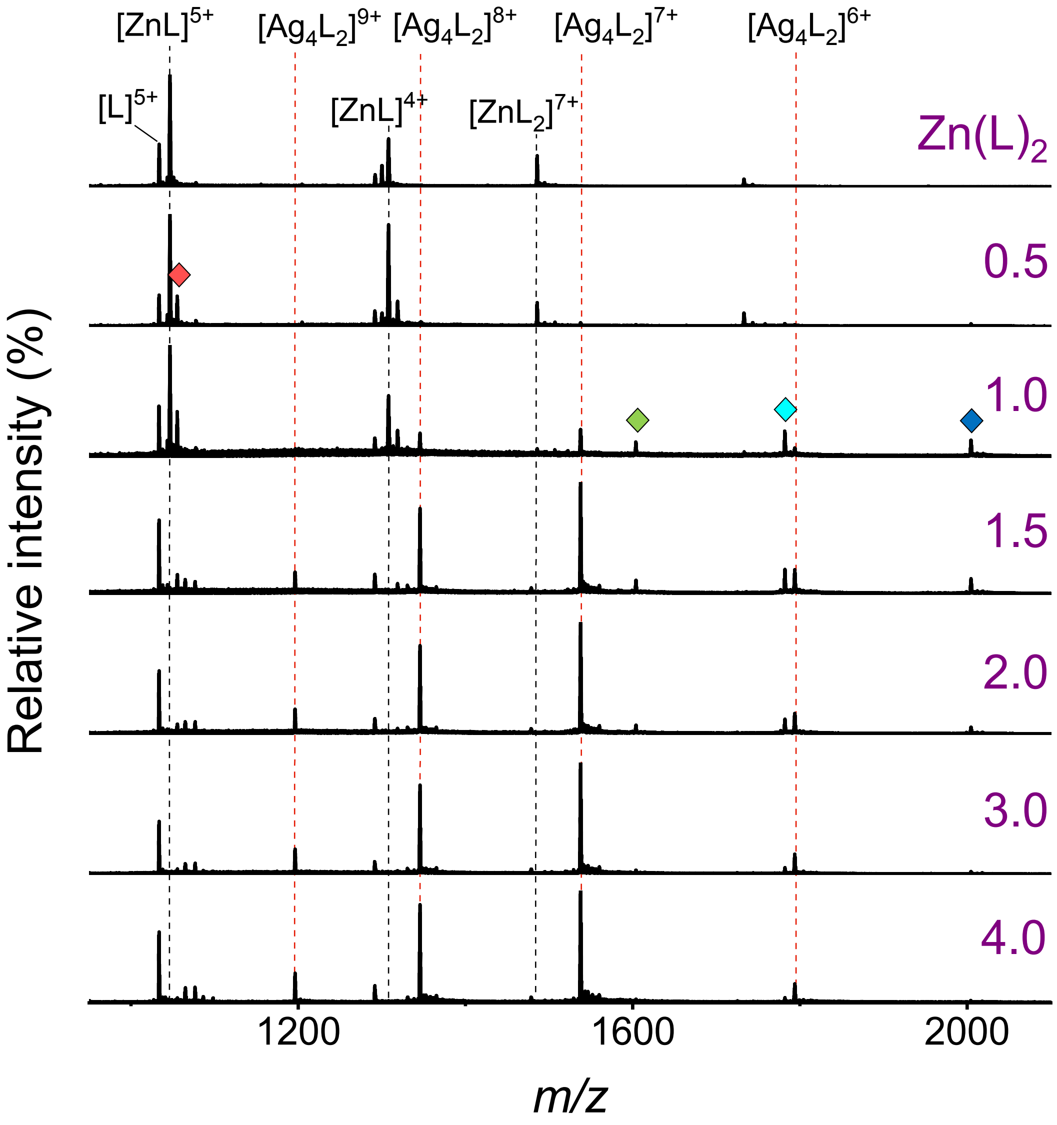

Figure S9. (+)ESI-MS-monitored Ag(I) binding to $Zn(Hk45)_2$. Mass spectra of 2 µM $Zn(Hk45)_2$ complex solutions were recorded for 0-4.0 mol. eq. of Ag(I) (violet color) in 50 mM ammonium acetate, pH 7.4. Black and red dashed lines represent ZnL or ZnL_2 (derived from $Zn(Hk45)_2$) and Ag_4L_2 ions as the substrate the and final product, respectively. Red, green, cyan, and blue labels denote $[AgL]^{5+}$, $[Ag_5L_3]^{10+}$, $[Ag_5L_3]^{9+}$, and $[Ag_5L_3]^{8+}$ species, respectively. Other assigned signals are listed in Table S4.

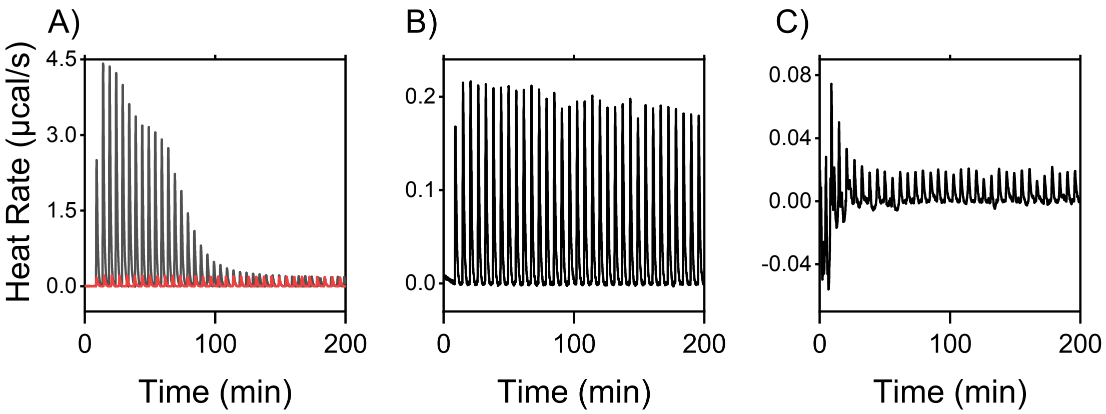

Figure S10. ITC analysis of Ag(I) binding to Hk14. Graphs show the baseline-subtracted thermogram. (A) ITC profile for titration of $AgNO_3$ into Hk14 with juxtaposed control titration of $AgNO_3$ into the buffer; (B) control titration of Ag(I) into buffer; (C) and titration of buffer into Hk14.

Table S1. The assigned m/z signals observed in MS-monitored Hk14 titration with $AgNO_3$ with the corresponding monoisotopic masses. The predominant $Ag_x(Hk14)_y$ complexes are marked in bold, where L denotes Hk14 monomer. The associated MS spectra are shown in Figure 8.

Ag(I) eq.	Observed m/z values	Calculated mass (Da)	$Ag_x(Hk14)_y$
0	759.38 2+	1516.75	L
0.5	812.33 2+	1622.66	**AgL**
	865.28 2+	1728.56	Ag_2L
	1082.78 3+	3245.34	Ag_2L_2
	1118.07 3+	3351.21	Ag_3L_2
	1217.99 4+	4867.96	Ag_3L_3
1.0	812.33 2+	1622.66	**AgL**
	865.28 2+	1728.56	Ag_2L
	1082.77 3+	3245.31	Ag_2L_2
	1118.07 3+	3351.21	Ag_3L_2
	1153.37 3+	3457.11	Ag_4L_2
	1217.98 4+	4867.92	Ag_3L_3
	1244.45 4+	4973.80	Ag_4L_3
	1017.15 5+, 1270.94 4+	5079.76	Ag_5L_3
	1341.48 5+	6702.4	Ag_6L_4
1.5	812.33 2+	1622.66	**AgL**
	865.27 4+, 1153.36 3+	3457.08	**Ag_4L_2**
	1016.95 5+, 1270.93 4+,	5079.72	**Ag_5L_3**
	1341.45 5+	6702.25	Ag_6L_4
2.0	865.28 4+, 1153.37 3+	3457.11	**Ag_4L_2**
	1270.95 4+	5079.80	Ag_5L_3
3.0	865.28 4+, 1153.37 3+	3457.11	**Ag_4L_2**
	1270.96 3+	5079.84	Ag_5L_3
4.0	865.29 4+, 1153.38 3+	3457.14	**Ag_4L_2**

Table S2. The assigned m/z signals observed in MS-monitored $Zn(Hk14)_2$ titration with $AgNO_3$ and the corresponding monoisotopic masses. The predominant $Ag_x(Hk14)_y$ complexes are marked in bold, where L denotes Hk14 monomer. The associated MS spectra are shown in Figure 8.

Ag(I) eq.	Observed m/z values	Calculated mass (Da)	$Ag_x(Hk14)_y$
0	759.40 2+, 1517.79 1+	1516.79	L
	790.36 2+	1578.72	ZnL
	1032.83 3+	3095.49	ZnL_2
0.5	812.35 2+	1622.70	**AgL**
	865.31 2+	1728.62	Ag_2L
	1082.80 3+	3245.40	Ag_2L_2
1.0	813.35 2+	1622.70	**AgL**
	865.30 2+	1729.58	Ag_2L
	1082.80 3+	3245.40	Ag_2L_2
	1153.40 3+	3457.17	Ag_4L_2
	1270.94 4+	5079.76	Ag_5L_3
1.5	813.35 2+	1622.70	**AgL**
	865.30 2+,	1729.58	Ag_2L
	865.29 4+, 1153.39 3+	3457.17	**Ag_4L_2**
	1082.79 3+	3245.37	Ag_2L_2
	1270.97 4+	5079.88	**Ag_5L_3**
2.0	813.35 2+	1622.70	**AgL**
	865.30 4+, 1153.39 3+	3457.17	**Ag_4L_2**
	1270.97 4+	5079.88	**Ag_5L_3**
3.0	865.30 4+, 1153.40 3+	3457.20	**Ag_4L_2**
	1270.97 4+	5079.88	Ag_5L_3
4.0	865.30 4+, 1153.39 3+	3457.17	**Ag_4L_2**
	1270.98 4+	5079.92	Ag_5L_3

Table S3. The assigned m/z signals observed in MS-monitored Hk45 titration with $AgNO_3$ with the corresponding monoisotopic masses. The predominant $Ag_x(Hk45)_y$ complexes are marked in bold, where L denotes Hk45 monomer.

Ag(I) eq.	Observed m/z values	Calculated mass (Da)	$Ag_x(Hk45)_y$
0	861.30 6+, 1033.35 5+, 1291.44 4+	5161.80	L
0.5	878.95 6+, 1054.54 5+, 1317.91 4+<br>1318.04 8+, 1506.33 7+	5267.70<br>10536.32	**AgL**<br>Ag_2L_2
1.0	878.95 6+, 1054.54 5+, 1317.91 4+<br>1344.39 8+<br>1318.05 8+, 1506.32 7+	5267.65<br>10747.12<br>10536.40	**AgL**<br>Ag_2L<br>Ag_2L_2
1.5	878.95 6+, 1054.53 5+, 1317.92 4+<br>896.60 6+, 1075.73 5+<br>1195.69 9+, 1344.67 8+, 1536.74 7+<br>1603.27 10+, 1781.08 9+, 2003.37 8+	5267.65<br>5373.65<br>10750.18<br>16018.96	**AgL**<br>Ag_2L<br>**Ag_4L_2**<br>**Ag_5L_3**
2.0	878.80 6+, 1054.54 5+, 1317.92 4+<br>896.60 6+, 1075.73 5+<br>1195.46 9+, 1344.52 8+, 1536.44 7+<br>1603.18 10+, 1780.97 9+, 2003.37 8+	5267.70<br>5373.65<br>10748.08<br>16018.96	AgL<br>Ag_2L<br>**Ag_4L_2**<br>Ag_5L_3
3.0	878.95 6+, 1054.54 5+, 1317.90 4+<br>1195.46 9+, 1344.64 8+, 1536.44 7+<br>1603.18 10+, 1780.99 9+, 2003.72 8+	5267.70<br>10748.08<br>16021.76	AgL<br>**Ag_4L_2**<br>Ag_5L_3
4.0	1195.21 9+, 1344.64 8+, 1536.40 7+<br>1781.15 9+, 2003.72 8+	10747.80<br>16021.76	**Ag_4L_2**<br>Ag_5L_3

Table S4. The assigned m/z signals observed in MS-monitored $Zn(Hk45)_2$ titration with $AgNO_3$ with the corresponding monoisotopic masses. The predominant $Ag_x(Hk45)_y$ complexes are marked in bold, where L denotes Hk45 monomer.

Ag(I) eq.	Observed m/z values	Calculated mass (Da)	$Ag_x(Hk45)_y$
0	861.28 6+, 1033.35 5+	5161.75	L
	871.63 6+, 1045.75 5+, 1306.94 4+	5223.76	ZnL
	1155.18 9+, 1299.19 8+, 1484.66 7+	10385.62	ZnL_2
0.5	878.96 6+, 1054.55 5+, 1317. 94 4+	5267.55	**AgL**
	1318.28 8+, 1506.17 7+	10536.19	Ag_2L_2
	1536.91 7+	10751.37	Ag_4L_2
	1781.10 9+, 2003.74 8+	16020.90	Ag_5L_3
1.0	878.96 6+, 1054.54 5+, 1317. 68 4+	5267.70	AgL
	1344.69 8+, 1536.62 7+,1792.90 6+	10749.34	Ag_4L_2
	1603.20 10+, 1780.92 9+, 2003.63 8+	16019.28	Ag_5L_3
1.5	878.93 6+, 1054.52 5+	5267.60	AgL
	1075.75 5+	5373.75	Ag_2L
	1195.44 9+, 1344.61 8+, 1536.55 7+,	10749.34	$\mathbf{Ag_4L_2}$
	1603.15 10+, 1780.93 9+, 2003.37 8+	16018.96	Ag_5L_3
2.0	878.96 6+, 1054.55 5+	5267.55	AgL
	1075.74 5+	5373.70	Ag_2L
	1195.44 9+, 1344.67 8+, 1536.61 7+,	10749.34	$\mathbf{Ag_4L_2}$
	1603.09 10+, 1780.89 9+, 2003.37 8+	16018.96	Ag_5L_3
3.0	1054.55 5+	5267.55	AgL
	896.60 6+, 1075.73 5+	5373.65	Ag_2L
	1195.48 9+, 1344.67 8+, 1536.61 7+,	10749.34	$\mathbf{Ag_4L_2}$
	1603.09 10+, 1780.89 9+, 2003.50 8+	16019.01	Ag_5L_3
4.0	896.62 6+, 1075.74 5+	5373.70	Ag_2L
	1096.93 5+	5479.65	Ag_3L
	1195.44 9+, 1344.66 8+, 1536.62 7+,	10749.34	$\mathbf{Ag_4L_2}$
	1781.00 9+	16020.00	Ag_5L_3

Table S5. Thermodynamic parameters of Ag(I) complexation with Hk14 peptide derived from fitting the data to a multiple sites model in $AgNO_3$ titration to Hk14 and a reverse titration of Hk14 to $AgNO_3$, and from an independent model in titration of $AgNO_3$ to the Hk14 in presence of 0.5 and 1 mol. eq. of $ZnSO_4$.

	Hk14 + Ag(I)	Zn(II):Hk14 = 1:1 + Ag(I)	Zn(II):Hk14 = 1:2 + Ag(I)	Ag(I) + Hk14
Model fitted	Multiple Sites, Blank (linear)	Independent, Blank (linear)	Independent, Blank (linear)	Multiple Sites
Hk14 (µM)	125 (analyte)	125 (analyte)	125 (analyte)	1080 (titrant)
Zn(II) (cell) (µM)	-	125	62.5	-
Ag(I) (µM)	5300 (titrant)	5300 (titrant)	5300 (titrant)	31 (analyte)
n_1	0.8 ± 0.01	1.9 ± 0.02	2.1 ± 0.02	0.4 ± 0.01
K_{d1} (nM)	5.7 ± 5.41	4059.0 ± 679.70	4097.0 ± 651.80	1.0 ± 0.34
ΔH_1 (kcal/mol)	-17.4 ± 0.11	-13.0 ± 0.23	-12.8 ± 0.21	-24.4 ± 0.72
$T\Delta S_1$ (kcal/mol)	-6.2	-5.6	-5.4	-12.1
n_2	1.3 ± 0.01	-	-	1.2 ± 0.06
K_{d2} (µM)	5.6 ± 0.40	-	-	0.4 ± 0.22
ΔH_2 (kcal/mol)	-13.0 ± 0.18	-	-	-5.4 ± 0.43
$T\Delta S_2$ (kcal/mol)	-5.9	-	-	3.3
intercept (µcal)	-13.6 ± 1.22	-13.6 ± 1.22 (fixed)[a]	-13.6 ± 1.22 (fixed)[a]	-
slope	0.05 ± 0.022	0.05 ± 0.022 (fixed)[a]	0.05 ± 0.022 fixed)[a]	-

[a] Blank linear model was fixed. The parameters of the model are equal to the parameters obtained from the Hk14 + Ag(I) titration.

References:

1. Padjasek, M.; Kocyła, A.; Kluska, K.; Kerber, O.; Tran, J. B.; Krężel, A., Structural zinc binding sites shaped for greater works: Structure-function relations in classical zinc finger, hook and clasp domains, *J. Inorg. Biochem.* 2020, *204*: 110955. Doi: 10.1016/j.jinorgbio.2019.110955
2. Eyer, P.; Worek, F.; Kiderlen, D.; Sinko, G.; Stuglin, A.; Simeon-Rudolf, V.; Reiner E. Molar absorption coefficients for the reduced Ellman reagent: reassessment, *Anal. Biochem*. 2003, *312*, 224–227. Doi: 10.1016/s0003-2697(02)00506-7.
